# Residential energy efficiency interventions: A meta‐analysis of effectiveness studies

**DOI:** 10.1002/cl2.1206

**Published:** 2021-12-17

**Authors:** Miriam Berretta, Joshua Furgeson, Yue (Nicole) Wu, Collins Zamawe, Ian Hamilton, John Eyers

**Affiliations:** ^1^ International Initiative for Impact Evaluation (3ie) London UK; ^2^ Independent Consultant, 3ie Hong Kong China; ^3^ Independent consultant, 3ie Lilongwe Malawi; ^4^ UCL Energy Institute London UK

## Abstract

**Background:**

The residential sector releases around 17% of global greenhouse gas emissions and making residential buildings more energy efficient can help mitigate climate change. Engineering models are often used to predict the effects of residential energy efficiency interventions (REEI) on energy consumption, but empirical studies find that these models often over‐estimate the actual impact of REEI installation. Different empirical studies often estimate different impacts for the same REEI, possibly due to variations in implementation, climate and population. Funding for this systematic review was provided by the evaluation function at the European Investment Bank Group.

**Objectives:**

The review aims to assess the effectiveness of installing REEIs on the following primary outcomes: energy consumption, energy affordability, CO_2_ emissions and air quality indices and pollution levels.

**Search Methods:**

We searched CAB Abst, Econlit, Greenfile, Repec, Academic Search Complete, WB e‐lib, WoS (SCI and SSCI) and other 42 databases in November 2020. In addition, we searched for grey literature on websites, checked the reference lists of included studies and relevant reviews, used Google Scholar to identify studies citing included studies, and contacted the authors of studies for any ongoing and unpublished studies. We retrieved a total of 13,629 studies that we screened at title and abstract level, followed by full‐text screening and data extraction.

**Selection Criteria:**

We included randomised control trials, and quasi‐experimental studies that evaluated the impact of installing REEIs anywhere in the world and with any comparison.

**Data Collection and Analysis:**

Two independent reviewers screened studies for eligibility, extracted data and assessed risk of bias. When more than one included study examined the same installation of the same type of REEI for a similar outcome, we conducted a meta‐analysis. We also performed subgroup analyses.

**Main Results:**

A total of 16 studies were eligible and included in the review: two studies evaluated the installation of efficient lighting, three studies the installation of attic/loft insulation, two studies the installation of efficient heat pumps, eight studies the installation of a bundle of energy efficiency measures (EEMs), and one study evaluated other EEMs. Two studies, neither appraised as having a low risk of bias, find that lighting interventions lead to a significant reduction in electricity energy consumption (Hedges' *g* = −0.29; 95% confidence interval [CI]: −0.48, −0.10). All the other interventions involved heating or cooling, and effects were synthesizised by warmer or colder climate and then across climates. Four studies examined the impact of attic/loft insulation on energy consumption, and two of these studies were appraised as having a low risk of bias. Three studies took place in colder climates with gas consumption as an outcome, and one study took place in a warmer climate, with the electricity consumption (air conditioning) as the outcome. The average impact across all climates was small (Hedges' *g* = 0.04; 95% CI: −0.09, 0.01) and statistically insignificant. However, two of the studies appear to have evaluated the effect of installing small amounts (less than 75 mm) of insulation. The other two studies, one of which was appraised as low risk of bias and the other involving air conditioning, found significant reductions in consumption. Two studies examined the impact of installing electric heat pumps. The average impact across studies was not statistically significant (Hedges' *g* = −0.11; 95% CI: −0.41, 0.20). However, there was substantial variation between the two studies. Replacing older pumps with more efficient versions significantly reduced electricity consumption in a colder climate (Hedges' *g* = −0.36; 95% CI, −0.57, −0.14) in a high risk of bias study. However, a low risk of bias study found a significant increase in electricity consumption from installing new heat pumps (Hedges' *g* = 0.09; 95% CI, 0.06, 0.12). Supplemental analyses in the latter study indicate that households also used the heat pumps for cooling and that the installed heat pumps most likely reduced overall energy consumption across all sources—that is, households used more electricity but less gas, wood and coal. Seven studies examined bundled REEIs where the households chose which EEMs to install (in five studies the installation occurred after an energy audit that recommended which EEMs to install). Overall, the studies estimated that installing an REEI bundle is associated with a significant reduction in energy consumption (Hedges' *g* = −0.36; 95% CI, −0.52, −0.19). In the two low risk of bias studies, conducted with mostly low‐income households, installed bundles reduced energy consumption by a statistically significant amount (Hedges' *g* = −0.16; 95% CI, −0.13, −0.18).

**Authors' Conclusions:**

The 16 included studies indicate that installing REEIs can significantly reduce energy consumption. However, the same type of REEI installed in different studies caused different effects, indicating that effects are conditional on implementation and context. Exploring causes of this variation is usually not feasible because existing research often does not clearly report the features of installed interventions. Additional high quality impact evaluations should be commissioned in more diverse contexts (only one study was conducted in either Asia or Africa—both involved lighting interventions—and no studies were conducted in South America or Southern Europe).

## 1 PLAIN LANGUAGE SUMMARY

### 1.1 The review in brief

The installation of energy efficiency measures (EEMs) in residential buildings reduces energy consumption, however, the evidence is limited and the risk of bias of the included studies often high. These results must be used with caution and more high‐quality impact evaluations in the field are needed.

### 1.2 What is this review about?

One of the key ways to mitigating climate change is by improving energy efficiency that can help to reduce energy consumption. Making housing more efficient presents a clear opportunity, as the residential sector releases around 17% of global emissions.

Engineering models indicate that residential energy consumption, and the associated CO_2_ emissions, could be reduced by installing residential energy efficiency interventions (REEIs). Yet studies that examine the actual impact of EEMs often find these models too optimistic about reductions in consumption.

This SR synthesises impact evaluations to estimate the average effects of installing different EEMs on energy consumption and examines how that effect differs across contexts and population subgroups. This study aims to provide useful information to inform energy strategy and policy design, implementation and financing decisions.

### 1.3 What studies are included?

The review includes studies with an experimental or quasi‐experimental design that estimate the effect of installing EEMs on relevant outcomes. We identified 16 studies, most of which were implemented in high‐income countries, in particular United States and Europe.

### 1.4 What are the main findings of this review?

#### 1.4.1 What is the effect of installing EEMs on energy consumption?

Our synthesis finds promising evidence that installing EEMs bundles reduces energy consumption. On average, installing bundles significantly reduced energy consumption. In most studies, installing individual EEMs caused smaller, statistically significant reductions in consumption, but a few studies estimate larger or negligible changes and one study found an increase in consumption. The results were similar when focusing on the five low risk of bias studies, with the caveat that the high quality evidence examining any EEMs is limited to one or two studies. Currently, there is not enough evidence to formally rate EEMs effectiveness; only one or two low risk of bias studies examine each EEMs. The effectiveness of each EEMs depends on many contextual factors (such as implementation or specific EEM features), and existing studies do not rigorously compare EEMs to each other.

#### 1.4.2 What is the available evidence on funding mechanisms and costs?

All the interventions were fully or partially funded by governments, universities, or a mix of them. Eight studies conducted some type of cost analysis such as cost‐benefit or cost‐effectiveness analysis. Whilst some studies found that the energy saved by EEMs installation was greater than the installation cost, other studies identified small or even negative cost‐effectiveness results. Among the two low risk of bias studies, one found a small negative rate of return from installing an EEM bundle—primarily because the reductions in energy consumption were much smaller than expected—and the second found a large positive rate of return from installing attic insulation.

### 1.5 What do the findings of this review mean?

The results suggest that the installation of EEMs is effective, but the available rigorous evidence is limited. Careful consideration of EEMs features and context is important, as studies indicate that the same EEMs implemented in different ways can cause different impacts. EEMs impacts on energy consumption are not always straightforward, as households might use some EEMs to increase indoor comfort or shift from one energy source to another, resulting in more energy consumed.

In the future, EEMs funders and installers should incorporate empirical findings to improve forecasting of how EEMs and programmes actually impact energy consumption. In particular, future studies might examine the possible causes of the variation of impact, which has been observed among studies. Studies might look at how factors, such as preinstallation audits or government regulations, moderate EEMs' impact.

To understand and compare impacts, studies must precisely describe baseline conditions and implemented interventions, such as the amount of insulation installed and the efficiency ratings of original and replacement boilers.

Finally, studies should examine EEMs' impact in more diverse contexts such as Asia, Africa, South America or Southern Europe.

### 1.6 How up‐to‐date is this review?

The search was conducted in November 2020 and this Campbell Systematic Review is expected to be published in December 2021.

## 2 BACKGROUND

### 2.1 The problem, condition or issue

Scientists agree that human activities are causing widespread climate change, and that reducing carbon dioxide (CO_2_) and other greenhouse gas emissions is crucial to mitigating the global environmental and health threats caused by climate change (IPCC, 2021). For example, the Intergovernmental Panel on Climate Change (IPCC) recently found that limiting global warming to 1.5°C—the level necessary to reduce challenging impacts on ecosystems, human health, and well‐being—requires large emissions reductions and comprehensive social changes (IPCC, 2018).

Residential energy use creates substantial carbon emissions. The International Energy Agency (IEA) estimates that residential usage accounts for 22% of the overall global final energy use and 17% of emissions (IEA, 2019). In European countries, homes are responsible for between 25% and 30% of energy consumption and related carbon emissions (Eurostat, 2019; Itard & Meijer, 2008; Palmer & Cooper, 2013; SEAI, 2010). In residential buildings, roughly 32% of energy consumption is used for space heating, 29% for cooking, 24% for water heating, and the remainder (roughly 15%) by appliances, lighting, and cooling (Ürge‐Vorsatz et al., 2015).

Models predict that residential energy use, and the associated CO_2_ emissions, could be significantly reduced by installing REEIs (Gowrishankar & Levin, 2017, Russell‐Bennett et al., 2019). For example, one study reported that more energy efficient residential buildings could eliminate 550 million metric tons of CO_2_ equivalent emissions annually by 2050 compared to the reference case (1830; 38.1%) (Gowrishankar & Levin, 2017). In addition to reducing energy use and emissions, many REEIs are widely recognised as having the potential to improve health and well‐being, as well providing microeconomic and macroeconomic benefits (Campbell et al., 2014; Russell‐Bennett et al., 2019; Shrubsole et al., 2014). These REEIs could have a long life—the vast majority of existing dwellings will still be in use in 2050 (Mathiesen et al., 2016; Meijer et al., 2009).

Despite the promise of REEIs, a recent review of four studies found that REEIs saved less energy than forecasted (J‐PAL, 2019). Currently, there is no conclusive evidence on how installing REEIs affects energy consumption and ultimately global emissions. Synthesising the available evidence on REEIs will provide useful information to inform energy strategy and policy design, implementation and financing decisions.

### 2.2 The intervention

Improved residential energy efficiency can be achieved through flexible strategies, such as the installation of insulation, heating and lighting upgrades, boiler replacements, and new windows (GABC/IEA/UNEP, 2020). REEI installation can involve improvements in the building/dwelling envelope; upgrades in the technical building/dwelling systems, such as space heating and cooling (Filippidou et al., 2019); or mechanisms that facilitate the installation of REEIs and their correct use. The European Investment Bank (EIB) invests in projects designed to install such REEIs.

In this review, we focus on the installation of EEMs in residential settings, where residences include private or social houses such as blocks of flats (also known as apartment and/or condominium buildings), public housing, as well as single family detached or semi‐detached housing. The type of residence can affect both REEI installation and energy consumption. Owners of rental property are less likely to install REEIs unless they can charge higher rents or installation is required by regulation, as tenants receive most benefits. Renters are also less likely to install REEIs as landlords typically do not allow property/equipment changes and renters usually stay for shorter periods and so are less likely to recoup REEI costs over time (Palmer & Cooper, 2013). In addition, rentals that include utilities with the rent typically consume more energy (Leth‐Petersen & Togeby, 2001).

REEIs refer to the installation of EEMs that alter the residential building/dwelling, as well as complementary interventions that aim to increase the uptake and persistence of EEMs, such as provision of information aimed at making a better use of the technology (Russell‐Bennett et al., 2019; Willand et al., 2015). Many REEIs involve installing multiple EEMs, such as attic insulation and new windows, as well as replacing the boiler or furnace. Governments and other organisations often fully or partially subsidise interventions for low income households and sometimes the broader housing market (Jacobsen et al., 2012). In this synthesis, we focus on two types of REEIs: EEM installation with and without behavioural interventions.

#### 2.2.1 EEM installation

EEM installation includes the replacement and upgrades of heating and cooling systems, the installation of insulation, more efficient boilers and heating, ventilation, and air conditioning technologies, among others (EEM installation examples are included in Adan & Fuerst, 2016; Howden‐Chapman et al., 2007; Maher, 2013). EEM installation often involves “weatherisation” which increases energy efficiency by protecting the building from sunlight, wind and precipitation (examples of studies evaluating EEM installations are Fowlie et al., 2018; Francisco et al., 2017; Pigg et al., 2018). EEMs can be further categorised by the amount of postinstallation household involvement required:

- *Passive* measures, such as insulation, do not require households to adopt a particular behaviour once completed
- *Semi‐passive* measures, for instance upgraded windows and doors, require residents to follow some simple behaviours (for instance, closing windows and doors to keep the rooms warm/cool)
- *Active* measures require continued correct behaviour for effectiveness, for instance heating controls.

EEMs are often installed after energy audits, which provide households with recommendations on appropriate REEIs, as well as information on applicable utility and state incentives that can reduce or eliminate the cost of installation (Taylor et al., 2014). By providing households with additional information, such as a simulation of benefits, audits can overcome informational barriers to installing EEMs.

#### 2.2.2 EEM installation combined with information provision interventions

These bundled interventions combine EEM installation with interventions that provide information designed to change household behaviour. These interventions inform households on how to best use the installed EEMs, such as advising households on how to set thermostats or how to reduce air conditioning load (examples of studies evaluating EEM installation in combination with behavioural interventions are James & Ambrose, 2017; Zivin et al., 2015). This guidance can be provided, for instance, by energy audits or other forms of technical assistance. Such guidance can be especially impactful for semi‐active and active EEMs. Behavioural interventions can be broader than information provision, but we limited this review to information provision because another systematic review (SR) published this year (Khanna et al., 2021) is focused on broader behavioural interventions to reduce energy consumption.

### 2.3 How the intervention might work

**Figure 1:**
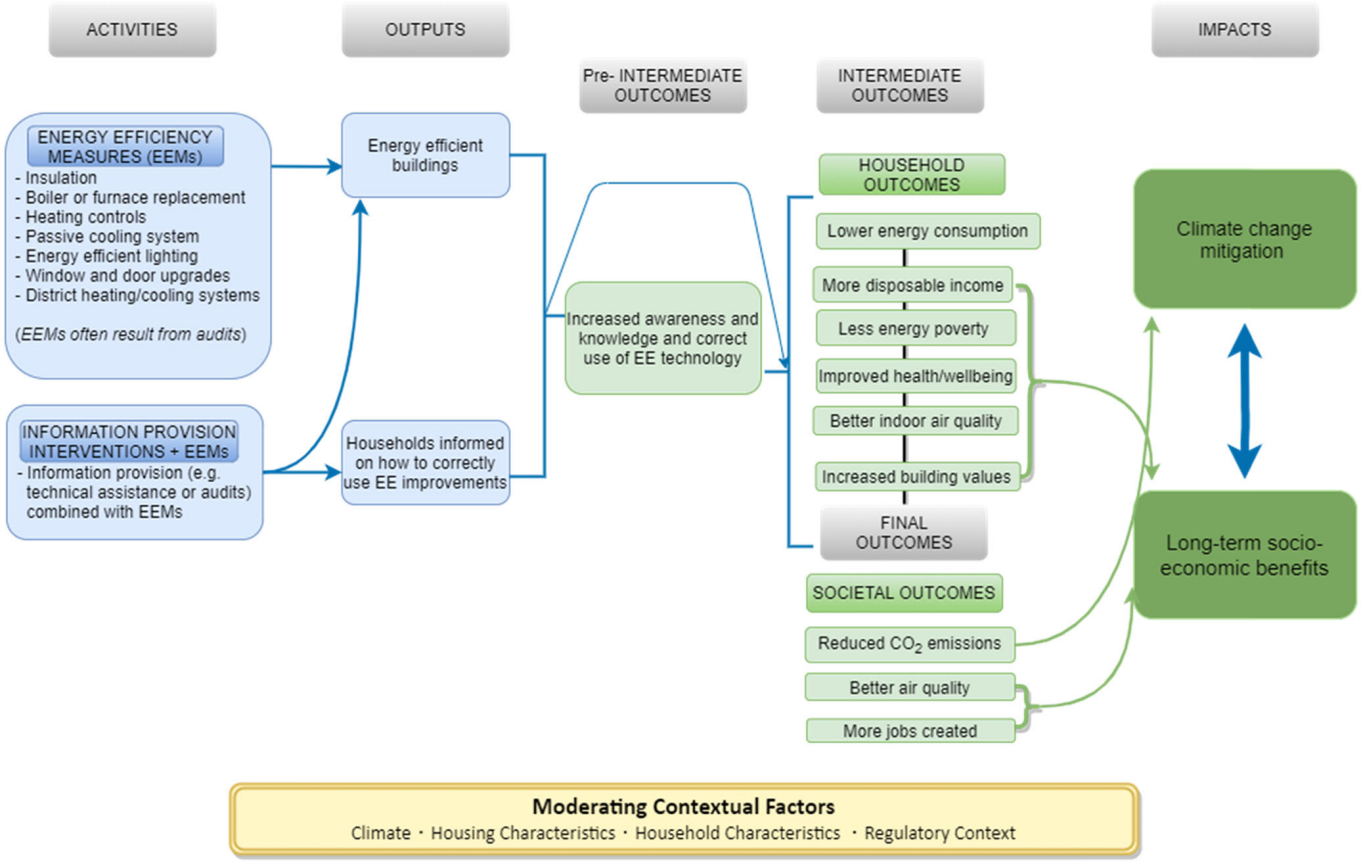
Theory of change. *Source*: 3ie, authors

After consulting relevant literature and experts, the review team developed a theory of change that proposes how REEIs in single‐ and multi‐family buildings can lead to climate change mitigation and long‐term socioeconomic benefits (Figure 1).

Starting from the left side of Figure 1, *activities* list the interventions that will be studied in this review: the installation of EEMs with and without information provision interventions. EEMs can be installed by the house's owner or as part of programme that subsidises the installation of one or multiple EEMs (Adan & Fuerst, 2016; Howden‐Chapman et al., 2017) (subsidisation is an REEI feature; we could not study the impact of this feature because almost all studies involved subsidies.) These installations often result from energy audits which identify relevant and cost‐effective upgrades (i.e., the audit can directly lead to EEMs). Audits can also provide guidance on how to use installed EEMs.

If the installation has been done correctly, the output should be a more energy‐efficient dwelling. When the intervention includes information provision, a household should also understand how the implemented EEMs work and how to correctly use them.

The preintermediate outcomes include increased knowledge and awareness of how to reduce energy consumption, and behavioural changes such as correctly using and maintaining the technologies. Information provision interventions aim to educate households, and the installation of EEMs can increase awareness and technical capabilities by having households use these technologies. Note that the preintermediate outcomes do not necessarily lead to the intermediate/final outcomes as, in some cases, EEMs like insulation are completely passive, and so the outputs lead directly to the intermediate/final outcomes.

In this theory of change, we have categorised intermediate outcomes as occurring at the household level, and the final outcomes at the societal level. At the household level, interventions can reduce energy consumption and increase disposable income, which leads to less energy poverty (lack of access to sufficient energy). Thus, EEMs can allow households to maintain indoor temperatures at a more comfortable level, especially in winter, improving health and wellbeing (Hills, 2012; Thomson et al., 2013). In addition, interventions might lead to better indoor air quality due to, for instance, better ventilation systems (Campbell et al., 2014; Grey et al., 2017; James & Ambrose, 2017; Russell‐Bennett et al., 2019; Shrestha et al., 2019). Finally, improvements in EE increase the value of the building stock that is an incentive for the houses' owners to invest in energy efficiency (Campbell et al., 2014; Russell‐Bennett et al., 2019; Filippidou et al., 2019). This sequential process is displayed by vertical black lines between the listed outcomes in Figure 1.

At the societal level, REEIs can cause reductions in global CO_2_ emissions, improved outdoor air quality, and create more jobs trough the EEMs installation process (Campbell et al., 2014; Filippidou et al., 2019; Russell‐Bennett et al., 2019).

Ultimately, these outcomes can lead to two long‐term societal impacts. First, a reduction in greenhouse gas emissions due to lower energy consumption will help to mitigate climate change. Secondly, the rest of the outcomes such as less energy poverty, better health and better air quality, can lead to long‐term socioeconomic impacts which include increased well‐being, especially for low‐income households who have more disposable income; reduced burden on the health sector due to less air pollution and warmer homes in winter; fewer shocks on energy demand due to cold or hot weather; and direct and indirect effects on the economy through, for instance, increased GDP and increased tax revenues (Campbell et al., 2014).

#### 2.3.1 Moderating contextual factors

The effects of REEI installation can vary depending on the context (Russell‐Bennett et al., 2019), and accordingly the theory of change includes moderating factors. These include the characteristics of the housing (such as the age of the building), climate, the applicable policies and building standards, and the income level of the households. REEIs might have different impacts for low‐income households due to the correlation between household income and energy consumption, after controlling for building characteristics (Abrahamse & Steg, 2009; Santin et al., 2009).

Figure 1 presents the anticipated theory of change, but the installation of REEIs is a complex process involving many different actors (such as installers and beneficiaries), and consequently some REEIs might lead to higher energy consumption or impaired wellbeing (Bone et al., 2010; Shrubsole et al., 2014). For instance, simply adding insulation without adjusting ventilation can reduce air circulation and the additional moisture can lead to mould and increases in other indoor‐generated pollutants (Pigg et al., 2018; Shrubsole et al., 2014), or lead to overheating in summer (RAND, 2020). Similarly, installing REEIs might cause increased energy usage if households feel that their “good behaviour” allows increased energy consumption in other areas, so‐called moral licensing (Jacobsen et al., 2012; Tiefenbeck et al., 2013).

Finally, REEIs might increase energy consumption due to the “rebound effect” of affordability (Davis et al., 2014; Shrubsole et al., 2014). This happens when the installed EEMs: (a) reduce the cost of operating equipment, causing the equipment to be used more (*direct rebound effect*), or (b) EEMs save households money and households use part of the saved income to increase energy consumption (*indirect rebound effect*). Therefore, simply considering energy consumption might underestimate utility gains from implementing these interventions, hence it is important to understand the causes of an increase in energy consumption in each context (Allcott & Greenstone, 2012; Hong et al., 2009).

### 2.4 Why it is important to do this review

Large investments are being made in residential energy efficiency. In 2019, roughly US$150 billion was invested globally in energy efficiency in the overall building sector, which includes residences (IEA, 2020). The EIB invested €4.6 billion in energy efficiency projects in Europe and around the world in 2019 (EIB, 2020). Energy efficiency building upgrades are also a sector of interest to major climate change funders like the World Bank and other multilateral development banks. In 2018, U.S. utilities spent roughly US$14 billion on residential energy efficiency programmes (U.S. Energy Information Administration, 2020).

3ie recently conducted an *evidence gap map (EGM)* on energy efficiency interventions which identified a cluster of impact evaluations examining REEI interventions (Berretta et al., 2021). Several impact evaluations found that REEIs can reduce demand for electricity, natural gas and heating oil, and ultimately contribute to reduced emissions and improved health (see for instance Koirala et al., 2013; Maidment et al., 2014). However, the estimated effects varied across studies. This SR synthesises this diverse literature to estimate an average effect, and examines how that effect differs across context and subgroups. This information can inform energy efficient policies, strategies and investments globally.

The EGM also identified four SRs that covered REEIs (Lomas et al., 2018; Maidment et al., 2014; Munton et al., 2014; Willand et al., 2015), but each has limitations. Munton et al. and Willand et al. do not synthesise the effects reported in the included studies, but rather describe the evidence base and identify possible characteristics of effective interventions. The Maidment et al. review focuses on health outcomes and hence is limited in scope, and had methodological limitations mainly due to the lack of critical appraisal or any discussion of bias of the included papers. Because of their methodological limitations, the quality appraisal in the EGM did not result in “high confidence” in the findings of any of these SRs. Finally, Lomas et al. conducted a review of heating control interventions on energy savings and cost‐effectiveness including 67 primary studies, mainly from the UK and USA. However, the authors only look at heating controls and they did not synthesise the results statistically.

Two other recent SRs examining REEIs that were not available at the time of the EGM search also do not provide a comprehensive summary. Kerr and Winskel (2020) explored how public policy can encourage investment in energy efficient retrofits, but did not assess the effects of the interventions. Russell‐Bennett et al. (2019) explored how intervention characteristics (such as target population and design) influence REEI effectiveness in Australia. This review had important limitations: the literature search was not comprehensive and the authors did not describe their approach to risk of bias and data synthesis.

This review has been funded by the evaluation function at the EIB, and the focus aligns with the EIB's climate action and environmental sustainability priorities. Specifically, REEIs are one of the EIB's priority areas as described in the EIB Energy Lending Policy and closely linked to the European Commission's Renovation Wave Strategy announced in October 2020 (European Commission, 2020).

Given the high rates of investments and the policy prioritisation of REEIs (including by organisations such as the EIB, the IEA, and the World Bank Group), the synthesis gap of studies is problematic—policy and practice are not being informed by systematic evidence. This SR aims to fill that gap and provide insights to key policy questions on the effectiveness of installing EEMs.

## 3 OBJECTIVES

This review aims to identify, appraise and synthesise the evidence available on the effectiveness of REEI installations, including those bundled with information provision. The synthesis estimates the overall impact of these interventions and examines some possible causes of variation in impacts. We also assess the cost‐effectiveness of REEIs.

We aim to answer the following research questions:

1. What are the effects of installing REEIs on energy consumption, energy security, and pollution outcomes?
2. To what extent do these effects vary by population group and location?
3. For the included studies, what are the implementation, context, and funding mechanisms?
4. What evidence is available on programme costs and incremental cost effectiveness in the included studies?

## 4 METHODS

We have followed the Methodological Expectations of Campbell Collaboration Intervention Reviews (MECCIR) Conduct and Reporting Standards (2019a, 2019b) and our process was based on recognised guidelines for SRs of effectiveness in international development (Waddington et al., 2012).

To address research questions 1 to 2, we synthesised evidence provided in impact evaluation studies and, whenever possible, analysed its corresponding effect size data. This allowed us to provide estimates of average effects and heterogeneity of reported changes in outcomes measured within the pathways described in the theory of change.

To capture evidence on the context, implementation and funding mechanisms, and costs (questions 3–4) we have searched for additional reports linked to the included studies, and extract all the relevant data which have been summarized and used to understand the findings.

### 4.1 Criteria for considering studies for this review

#### 4.1.1 Types of studies

To answer the first, second and fourth research questions, we included counterfactual studies that use an experimental or quasi‐experimental design and/or analysis method that can plausibly control for confounding and selection bias (i.e., different types of households choose to install REEIs and these differences, not the REEIs, impact outcomes).

Specifically, we included the following study types:

1. Randomised controlled trials with assignment at the individual, household, community or other cluster level, and quasi‐randomised trials using prospective methods of assignment such as alternation.
2. Nonrandomised designs with either a known assignment variable(s) or a seemingly random assignment process:
  a. Regression discontinuity designs, where assignment is based on a threshold measured before intervention, and the study uses prospective or retrospective approaches of analysis to control for unobservable confounding.
  b. Natural experiments with clearly defined intervention and comparison groups that exploit apparently random natural variation in assignment (such as a lottery) or random errors in implementation, and so forth.
3. Nonrandomised studies with pre and postintervention outcome data for both intervention and comparison groups, that use the following methods to control for confounding:
  a. Studies controlling for time‐invariant unobservable confounding, including difference‐in‐differences (such as models with an interaction term between time and intervention) and fixed‐effects models that include fixed effects for household and time.
  b. Studies assessing changes in outcome trends over a series of time points with a contemporaneous comparison group (controlled interrupted time series), and with sufficient observations to establish a trend and control for effects on outcomes due to factors other than the intervention (such as seasonality).
4. Nonrandomised studies involving a similar comparison group (including statistical matching, covariate matching, coarsened‐exact matching, propensity score matching) or control for confounding using multiple regression analysis. Because houses with similar physical characteristics can have very different levels of energy consumption (Arumägi and Kalamees, 2014; Summerfield et al., 2007), the matching or analysis must include a baseline measure of the outcome.
5. Nonrandomised studies that control for confounding using instrumental variable approaches such as two‐stage least squares estimation.

We refer to studies in categories 3 or 4 as *quasi‐experiments*.

For Research Question 3, we also looked at additional studies related to implementation, financial mechanisms and context for the studies included in the review.

#### 4.1.2 Types of participants

We included any study that involved households living in single‐family or multi‐family residential buildings (dwellings) regardless of income or geographic location.

We excluded studies that installed EEMs in public, commercial, office or industrial buildings because, whilst a priority of institutions such as the EIB, the EGM only identified three studies targeting public commercial, office or industrial buildings. When a study included residential and nonresidential buildings and reported separate estimates for residential buildings (e.g., Liang et al., 2018), the residential estimates are eligible for inclusion in this SR.

#### 4.1.3 Types of interventions

We included studies that measure the impact of at least one of the interventions listed in Table 1. Studies that compare an EEM control group to a bundle of EEM + information provision intervention group are not eligible because they are only examining the impact of information provision rather than the impact of an EEM plus the information provision However, studies that compare EEM + information provision intervention to a control group that does not receive any or another treatment, will be included.

**Table 1:** Eligible interventions

Category	Intervention
EEMs (interventions can be combined)	Wall/roof/floor cavity insulation
	Loft/attic insulation
	External/internal wall insulation
	Replacement (oil or gas) boiler or furnace or central air conditioning
	Heating controls
	Passive cooling system and design
	Energy efficient lighting (such as compact fluorescent light bulbs)
	Window and door upgrades
	District heating/cooling systems
Behavioural interventions + EEMs	Information provision + EE interventions

#### 4.1.4 Types of outcome measures

##### Primary outcomes

We included all studies that measured at least one of the primary outcomes listed in Table 2. The primary outcomes are: energy consumption, energy affordability, CO_2_ emissions and, air quality indices. Because the focus of the review is on the effect of EEM on outcomes linked to climate change, at least one of the primary outcomes must be reported for a study to be included.

**Table 2:** Eligible outcomes

Level	Outcome category	Description
Primary outcomes	Net energy savings or consumption changes	Actual savings in net energy (including fuel) or changes in energy consumption that are attributable to the EEM or REEI
	Energy security	The uninterrupted availability of energy at an affordable price
	GHG emissions	Actual carbon related emissions (CO_2_) and noncarbon related emissions, such as methane (CH_4_), nitrous oxide (N_2_O) and fluorinated gases
	Air quality indices	Actual air pollution from the combustion of fuels at an electrical power plant or from combustion of heating fuels, such as natural gas or fuel oil at a residence
Secondary outcomes	Income savings	Reduced expenditures due to more efficient new or upgraded equipment (e.g., bill savings)
	Health status, comfort, and wellbeing	Better health and quality of life resulting from the installation of EEMs
	Job creation	New job creation due to the installation of EEMs or otherwise attributed to use of EEMs
	Building stock value	Increased property value due to the installation of new equipment or renovation of equipment

As predictions of energy consumption can often be inaccurate (Fowlie et al., 2018; Gillingham et al., 2013; Grimes et al., 2016; Howden‐Chapman et al., 2007), studies must report actual energy consumption. We also exclude estimated GHG emissions and estimated income savings (see James & Ambrose, 2017), where study authors estimate these quantities by multiplying changes in measured energy consumption by a factor (such as 29 cents/kWh) because differences between studies might be due to different factors.

##### Secondary outcomes

Because EE interventions have multiple benefits (Campbell et al., 2014), we also included secondary outcomes in health, well‐being, economics, and behavioural outcomes for those studies that include at least one of the primary outcomes.

#### 4.1.5 Duration of follow‐up

We included any follow‐up duration, coding multiple outcomes if studies report multiple follow‐ups.

#### 4.1.6 Types of settings

We accepted studies from any type of setting and any part of the world. We only reviewed studies conducted in real‐world settings (i.e., we did not include efficacy studies).

### 4.2 Search methods for identification of studies

To reduce the risk of publication bias and identify relevant evidence, we conducted a comprehensive search for published and unpublished studies in November 2020, adopting a detailed search strategy reported in Supporting Information Appendix C.

REEIs have improved incrementally and constantly over time. To include interventions most similar to those being implemented now, the search was limited to studies published on or after January 1, 2000.

No language restrictions were placed on the searches; however, all searches were conducted in English.

#### 4.2.1 Electronic searches

We conducted the search strategy in the following academic databases:

- CAB Abst
- Econlit
- Greenfile
- Repec
- Academic Search Complete
- WB e‐lib
- WoS (SCI & SSCI).

We also searched the organisational databases and evidence repositories listed in Supporting Information Appendix C.

#### 4.2.2 Searching other resources

We screened all studies listed in the bibliography of the energy efficiency EGM and other relevant SRs and literature reviews. In addition, we screened the reference lists of all included studies (backward citation search) and used Google Scholar to search for studies that cited included studies (forward citation search).

To identify additional studies, we contacted key experts and organisations through our review external advisory group and internal EIB reference group.

#### 4.2.3 Targeted search for studies addressing Q3

To answer Question 3 relating to implementation, financial mechanisms and context, we attempted to identify programme and project documents associated with the programmes identified in the first stage of the search. We did this by undertaking a targeted search for programme names and authors using Google, after we identified the studies included in the review. Evidence on context and mechanisms was collected from all the included studies. Information on programme mechanisms was either suggested by study authors or identified by the review team.

### 4.3 Data collection and analysis

#### 4.3.1 Criteria for determination of independent findings

Estimating standard meta‐analytic average effects assumes that each included effect is statistically independent (Hedges, 2019). The statistical significance of findings can also be inflated when there are dependencies within a study. Dependent effect sizes can arise when: (1) one study provides multiple results for a similar outcome of interest, (2) one study has multiple treatment arms compared to the same comparison group, or (3) multiple studies use the same data and report on the same outcome. We therefore used the following rules to ensure that only statistically independent effect sizes were included as primary findings (other effect sizes are reported in Supporting Information Appendix H).

When a study reported multiple outcomes using similar outcome constructs (Howden‐Chapman et al., 2007), to enhance the potential for meta‐analysis we selected the construct that is the most similar to other estimates for the same outcome type. For example, when studies included both measured and self‐reported energy consumption (Howden‐Chapman et al., 2007), for consistency across studies we extracted the measured consumption. When a study included more than one energy outcome (Adan & Fuerst, 2016; Fowlie et al., 2018; Grimes et al., 2016; James & Ambrose, 2017), such as electricity consumption and gas consumption and total energy (electricity + gas) consumption, we chose the outcome that would provide the most sensitive test of the intervention (such as gas for boilers or electricity for air conditioning).

No studies included more than one outcome period.

When we identified studies with multiple treatment arms and only one comparison group (Grimes et al., 2016; Hamilton et al., 2016; James & Ambrose, 2017; Suter & Shammin, 2013), we choose the intervention that most commonly resembles other studies' interventions as the primary comparison (other effect sizes are reported in Supporting Information Appendix H).

Several studies reported multiple effect size estimates using slightly different models; here we chose the one with the lowest assessed risk of bias or most similar to other studies. If a study included different analyses with overlapping samples (Alberini et al., 2019; Fowlie et al., 2018), we chose the one with the lowest risk of bias. Where we identified several studies/publications that report on the same analysis we used effect sizes from the most recent publication.

#### 4.3.2 Selection of studies

We imported all search results into EPPI‐Reviewer 4^1^ and removed duplicates. After testing the inclusion/exclusion criteria for operationalisability, two independent research assistants double screened all studies against the inclusion criteria using information available in the title and abstract; any disagreements were resolved through conversations with a core review team member. Where a study's title and abstract did not include sufficient information to determine relevance, the study was included for a full text review.

While undertaking title/abstract screening, we took advantage of the text‐mining capabilities of EPPI‐Reviewer 4, to reduce the initial screening workload (O'Mara‐Eves et al., 2015). We used the “Priority” screening function to prioritise screening the studies that were more likely to be eligible and accelerate the screening process. Ultimately, all the studies were independently reviewed by two screeners during the title and abstracts screening because we kept finding some potential includable studies until the end of the screening.

Studies included for full‐text screening were double screened by two independent reviewers. Disagreements were resolved by discussion with a core review team member and the input of an additional core reviewer if necessary.

The screening of studies for Question 3 took place later, after studies were identified for inclusion in the core effectiveness component of the review. The studies identified to answer Question 3 were assessed for relevance, that is, whether they (1) examined one of the programmes in an included effectiveness study, and (2) whether they provide information on the implementation processes, context or mechanisms at play.

#### 4.3.3 Data extraction and management

Using a standardised data extraction form (form provided in Supporting Information Appendix A), we extracted the following descriptive, methodological, and quantitative data from each included study:

- Descriptive data including authors and publication date, as well as other information to characterise the study including country, cost data, type of intervention and outcome, population, and context.
- Methodological information on study design, measurement and analysis methods, type of comparison (if relevant) and external validity (e.g., population and setting).
- Quantitative data for outcome measures, including outcome descriptive information, sample size in each intervention group, outcome means and SDs, test statistics (e.g., *t* test, *F* test, *p* values, 95% confidence intervals [CIs]), and so on.
- Information on interventions, including how the interventions was funded and with which financial mechanisms, transparency in conducting the study, household participation, contextual factors and programme mechanisms.

We extracted all data using Excel. Descriptive and qualitative data were double‐coded and checked by a core team member.

#### 4.3.4 Assessment of risk of bias in included studies

Our literature search was inclusive, and identified studies that did not undergo peer‐review. We assessed the risk of bias for the eligible impact evaluations, using the 3ie risk of bias tool (Supporting Information Appendix B) which covers both internal validity and statistical conclusion validity of experimental and quasi‐experimental designs (Waddington et al., 2012) and the bias domains and extensions to Cochrane's ROBINS‐I tool (Sterne et al., 2016).

Two reviewers independently assessed the risk of bias. When there were disagreements, they were resolved by discussion and the involvement of a senior reviewer. We conducted the risk of bias assessment at the study level, noting any potential differences in methods and the risk of bias for different outcomes.

We assessed the risk of bias based on the following criteria:

- Factors relating to baseline confounding and biases arising from differential selection into and out of the study (e.g., “Was any differential selection into or out of the study (attrition bias) adequately resolved?”);
- Factors relating to biases due to deviations from intended interventions (such as contamination) and motivational bias (Hawthorne effects);
- Factors relating to biases in outcomes data collection (such as social desirability, and recall bias);
- Factors relating to biases in reporting of analysis.

For each criterion, we coded each study as “Yes”, “Probably Yes”, “Probably No”, “No” and “No Information” according to how they address each domain. After the risk of bias was appraised for each criterion, an overall risk of bias rating was assigned using the following approach: (1) if any domain was appraised as “no” or “probably no”, then the overall risk of bias is high; (2) if all domains were appraised as “yes” or “probably yes”, then the overall risk of bias is low; (3) if the information needed to appraise one or more domain was unclear but the rest of the dimensions were appraised “yes” or “probably yes”, then the overall risk of bias is "some concerns".

#### 4.3.5 Measures of treatment effect

Studies examining similar outcomes might report effects using different metrics (e.g., some studies' outcomes are in kilowatt hours and others are in the natural logarithm of kilowatt hours). To enable a synthesis of these findings, all study effects have been converted to standardised effect sizes that express the magnitude or strength of the relationship between the intervention and outcome (Borenstein et al., 2009; Borenstein & Hedges, 2019).

For studies reporting difference‐in‐differences computed with means and SDs, we use the formula described in Morris (2008):

$$d=\frac{\left[\left(y_{1,t}-y_{0,t}\right)-\left(y_{1,c}-y_{0,c}\right)\right]}{\sqrt{\frac{\left(n_{t}-1\right)s_{1,t}^{2}+\left(n_{c}-1\right)s_{1,c}^{2}}{\left(n_{t}+n_{c}-2\right)}}},$$

where *y*_1,*t*_ and *y*_o,*t*_ are the post‐ and preintervention means for the treatment group, and *y*_1,*c*_ and *y*_o,*c*_ are the post‐ and preintervention means for the comparison group; *s*_1,*t*_ *s*_1,*c*_ are the postintervention sample SDs for the treatment and comparison groups, respectively; and *n*_*t*_ and *n*_*c*_ are the analytic sample sizes for the treatment and comparison groups, respectively.

$$V_{d}\text{size}=2\left(1-\rho \right)\times \left[1-\frac{3}{4N-9}\right]\times \left[\frac{n_{t}+n_{c}}{n_{t}n_{c}}\right]\times \left[\frac{n_{t}+n_{c}-2}{n_{t}{+n}_{c}-4}\right]+\left[1+\frac{d^{2}}{2(1-\rho )(\frac{n_{t}+n_{c}}{n_{t}n_{c}})}\right]-d^{2},$$

where ρ is the correlation between pre‐ and postintervention measures (based on a recommendation from our content expert, we assumed 0.75 for studies that did not report the correlation).

For studies reporting regression coefficients, we used formulae from Lipsey and Wilson (2001). Fowlie et al. (2018) report both an intent‐to‐treat (ITT) estimate and a complier average causal effect (CACE) estimated using two‐stage least squares. Because roughly 95% of treatment households did not install REEIs, the Fowlie ITT estimates a different impact than the average treatment‐on‐treated estimated by other studies; thus to calculate effect sizes we used the CACE and backed‐out the baseline SDs (Fowlie et al., 2018; table II). We used these SDs to compute the effect, instead of the outcome SD.

When the regression coefficient and the pooled SD of the outcome are available:

$$d=\frac{\beta_{t}}{S},$$

where $\beta_{t}$ is the coefficient on the treatment variable and

$$S=\sqrt{\frac{\left(n_{t}-1\right)s_{t}^{2}+\left(n_{c}-1\right)s_{c}^{2}}{\left(n_{t}+n_{c}-2\right)}}.$$

$$V_{d}=\frac{n_{t}+n_{c}}{n_{t}n_{c}}+\frac{d^{2}}{4(n_{t}+n_{c})}.$$

When studies do not report the outcome SD, we approximate a rough effect size using the coefficient *t* statistic. For the regression models that include covariates or fixed effects—almost of the models included in this study—the formulas make strong assumptions to approximate the effect size. Where the pooled SD of the outcome is unavailable but the sample size information is available for each group:

$$d=t\cdot \mathrm{stat}\sqrt{\frac{1}{n_{t}}+\frac{1}{n_{c}}}.$$

$$V_{d}=\frac{n_{t}+n_{c}}{n_{t}n_{c}}+\frac{d^{2}}{4(n_{t}+n_{c})}.$$

The *t* statistic (t‐stat) is calculated by dividing the coefficient by the standard error or using the reported t‐stat. If the authors do not report a t‐stat but report the *p* value to three decimal places, we used the Excel T.INV.2T function to approximate the *t* statistic.

Where the pooled SD and sample size of each group are unavailable, but the total sample size information is available, we used a formula that assumes both groups have identical sample sizes:

$$d=\frac{2t}{\sqrt{N}}.$$

$$V_{d}=\frac{4}{N}+\frac{d^{2}}{4N}.$$

For randomised trials reporting unadjusted odds ratios, we used the formula reported in Borenstein et al. (2011):

$$d=\frac{\ln(\mathrm{OR})\sqrt{3}}{\pi },$$

$$V_{d}=\frac{3\left[\frac{1}{T_{\mathrm{outcomes}}}+\frac{1}{C_{\mathrm{outcomes}}}+\frac{1}{T_{\mathrm{non}-\mathrm{outcomes}}}+\frac{1}{C_{\mathrm{non}-\mathrm{outcomes}}}\right]}{\pi^{2}},$$

where *T*_outcomes_ and *C*_outcomes_ are the number of participants having the outcomes for the treatment and control groups, respectively; and *T*_non‐outcomes_ and C_non‐outcomes_ are the number of participants not having the outcomes for the treatment and control groups, respectively.

We converted *d*'s to Hedge's *g* by multiplying by the following approximation: $1-\frac{3}{4N-9}$, and we converted *V_d_* to *V_g_* by multiplying by ${\left(1-\frac{3}{4N-9}\right)}^{2}$. To calculate SE_g_, we took the square root of *V_g_* (Borenstein et al., 2011).

We also calculated impacts in kWh by converting different metrics for energy consumption (m^3^ of gas consumed, 100 cubic feet of gas consumed, and 1,000,000 British thermal units) to kWh annually using the US Energy Information Administration's online energy conversion calculator. Some studies reported daily, monthly, or quadrimester energy consumption; we converted these to annual consumption by multiplying by 365, 12, and 3, respectively. For the five studies (Adan & Fuerst, 2016; Alberini et al., 2016; Alberini et al., 2019; Fowlie et al., 2018; Liang et al., 2018) that used the natural log of energy consumed as the outcome, we report the estimated *percentage change* in energy consumed as calculated by $e^{\beta }-1$.

#### 4.3.6 Unit of analysis issues

Unit of analysis issues arise when a study's unit of allocation (assignment) is different from the unit of analysis, and the analysis does not account for the potentially correlated outcomes of units within clusters. Only one included study (Carranza & Meeks, 2016) had a unit of assignment that differed from the unit of analysis. For this study, we use the author‐reported cluster‐corrected standard errors.

#### 4.3.7 Dealing with missing data

When studies did not provide data needed for meta‐analysis (such as means and SDs), we contacted two study authors to obtain the required information. One author (James & Ambrose, 2017) did not respond to the request and we were unable to obtain the necessary data. We excluded this study from the quantitative synthesis but included it in the descriptive analysis. Another author (Howden‐Chapman et al., 2007) did not respond to a request for data for three outcomes (Short Form‐36 full scales: role‐physical, role‐emotional, and social functioning); however, there was complete data for other outcomes and those outcomes have been included.

Two other authors (Carranza & Meeks, 2016; Maher, 2013) did not respond to a request for additional information needed to appraise risk of bias. These studies were appraised as *some concerns* for risk of bias.

#### 4.3.8 Assessment of reporting biases

We attempted to reduce publication bias by conducing a comprehensive search for all relevant studies and including grey literature in the review (see Vevea et al., 2019). In addition to the electronic searches, we performed backward and forward citation‐tracking, contacted experts, and searched websites. Although we had planned to conduct a funnel analysis, we did not due to the small number of studies in each meta‐analysis.

#### 4.3.9 Quantitative data synthesis

Once we identified all the eligible studies, we mapped out the interventions, climates, comparisons, and outcome measures. Based on an examination of these characteristics, we chose how best to synthesise findings across studies.

#### 4.3.10 Meta‐analysis

We only synthesised studies using meta‐analysis when we identified at least two effect sizes: (a) involving a similar intervention, and (b) implemented in a similar climate. We report separately by intervention category because we believe that funders, policymakers, installers, and households are interested in how specific EEMs impact energy consumption (not how any type of EEM impacts consumption). We analyse by climate because climate affects whether heating or cooling is mostly needed and how much heating or cooling is needed; climate thus directly determines how much energy consumption can be changed. Accordingly, we classify study climates by the average number of annual *heating degree days (HDD)* 1 a commonly used measure of the energy consumption required to *heat* buildings. HDDs measure how much (in degrees) and for how long (in days) the outside air temperature was *lower* than a specific *base temperature* (EU standard = 15.5°C). Specifically, we present impacts separately for warmer climates (300–830 average annual HDDs) and colder climates (1954–2860 average annual HDDs). James and Ambrose (2017) took place in a moderate climate (1000–1500 HDDs), but is not included in the meta‐analysis because the authors did not report sufficient information to calculate an effect size and the lead author did not respond to a request for this information. Some policymakers might be interested in an effect across climates, and so we also report an overall effect. We present the results this way because we believe our audience is interested in research questions such as:

- How does providing households with an energy audit and subsidising a tailored EEM bundle for the dwelling impact energy consumption in colder climates and in warmer climates?
- How does installing attic/loft insulation impact energy consumption in colder and warmer climates?
- How does providing heavily subsidised compact fluorescent lights impact electricity consumption?

Intervention characteristics, housing characteristics, and other relevant factors varied across studies, and so we conducted a maximum‐likelihood random‐effects meta‐analysis with inverse‐weighting by statistical precision using the *metafor* package in R (R Development Core Team 2018). The weights are based on within‐study statistical precision as well as the estimated between‐study variance. In case the estimates are sensitive to the estimator (Viechtbauer, 2005; Veroniki et al., 2016), Supporting Information Appendix G reports meta‐analysis statistics estimated using a restricted maximum likelihood estimator (Viechtbauer, 2005) and fixed‐effects meta‐analysis.

When there is only one study examining an intervention, we present the effect in a table and synthesise findings narratively.

#### 4.3.11 Subgroup analysis and heterogeneity reporting

For one type of intervention—EEM bundle—there were sufficient studies to conduct sub‐group meta‐analyses for the following categories of interest to the primary funder:

- Resident socioeconomic status
- Region of residency (European Union‐27 and the UK vs. Other)

We assess heterogeneity by calculating the *Q* statistic, *I* ^2^, and *τ* to provide an estimate of the amount of variability in the distribution of the true effect sizes (Borenstein et al., 2009). We complement this with a graphical presentation of heterogeneity of effect sizes using forest plots that include prediction intervals as recommended by Borenstein (2019).

#### 4.3.12 Sensitivity analysis

We conducted two sensitivity analyses. The first used the *leave1out* command in R to assess whether the results of the meta‐analysis were sensitive to the removal of any single study. For the meta‐analysis that included more than one low risk of bias study (EEM bundle), we also assessed sensitivity of results by removing high risk of bias studies from the meta‐analysis.

## 5 RESULTS

This section provides an overview of the included (and excluded) studies, including qualitative descriptions of interventions, sample populations, geographic coverage, and eligible outcome measures.

### 5.1 Description of studies

#### 5.1.1 Results of the search

**Figure 2:**
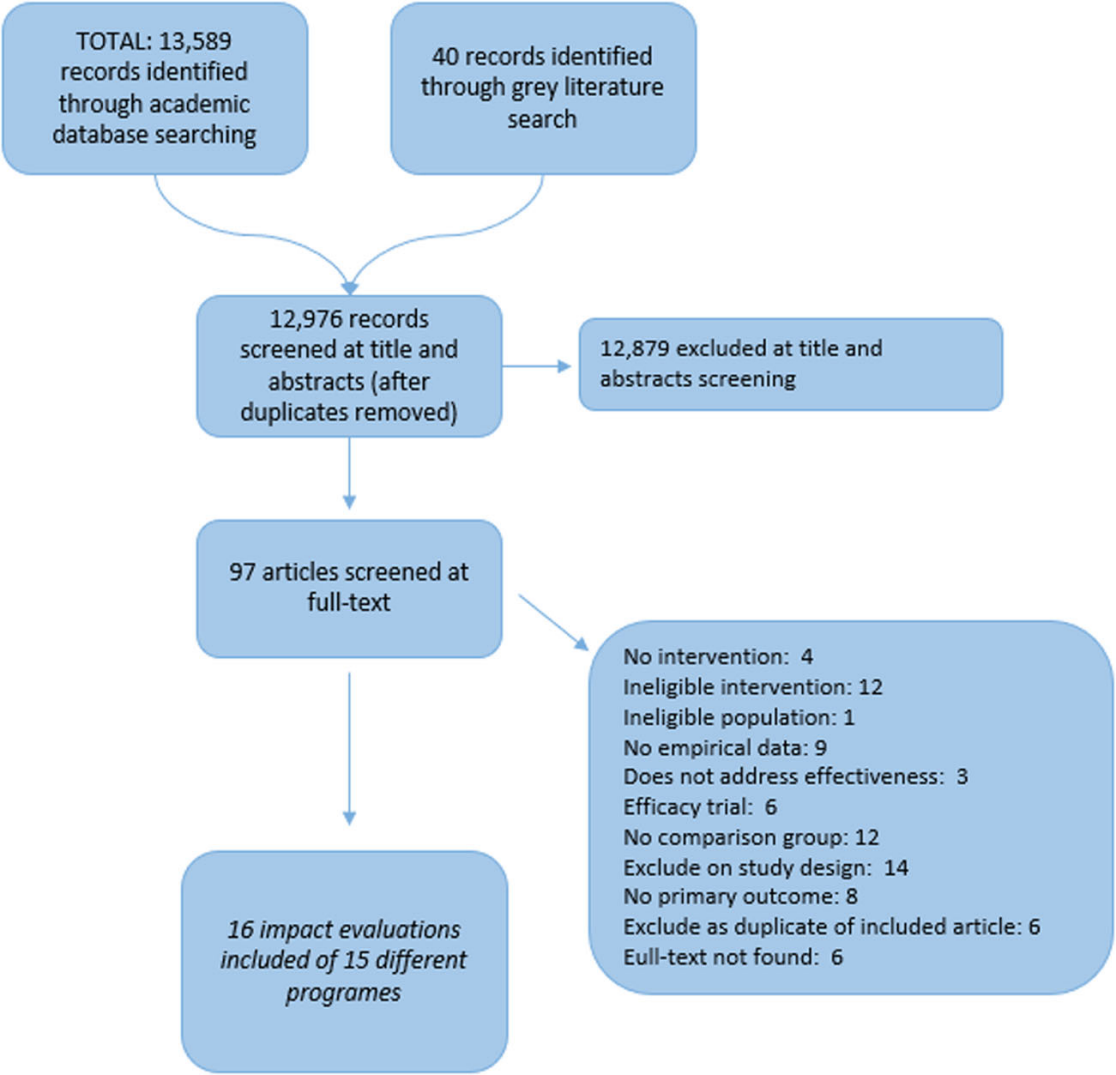
PRISMA flow‐chart

The PRISMA flow chart (Figure 2) shows the results of the search and screening processes conducted for this SR in November 2020 (although some relevant studies were identified through the EGM; Berretta et al., 2021), we conducted a new search tailored specifically to the inclusion criteria of this SR, and to include studies after the search for the EGM was conducted in April 2020.) The initial studies were identified by searching academic databases (*n* = 13,589) as well as by searching grey literature in websites, online libraries and repositories of selected organisations (*n* = 40). After removing duplicates, we screened 12,976 studies at the title and abstract level. We excluded 12,879 studies based on the inclusion criteria and 97 studies were identified as potentially relevant studies and underwent a full‐text screening. At the title and abstract screening, most of the studies were excluded because they did not include an intervention (6919), the intervention was not relevant (5015), the study design used was not one of those listed in the protocol (Berretta et al., 2021) (515), lack of empirical data (290), or they did not address effectivenss (131); the remaining studies were excluded because they were duplicates. Excluding a large number of the studies initially identified is not unusual. SRs often exclude the vast majority of studies identified through comprehensive searches (Wang et al., 2020).

During the full‐text screening stage, we excluded 81 studies for different reasons. Several studies were screened out for multiple reasons, but only the first reason was coded. The most common first reasons were: ineligible study design (14), the lack of a valid comparison group (12) or ineligible intervention (12 studies). The complete list of the studies excluded at the full‐text screening stage can be found at the end of this report.

#### 5.1.2 Geographic coverage

**Figure 3:**
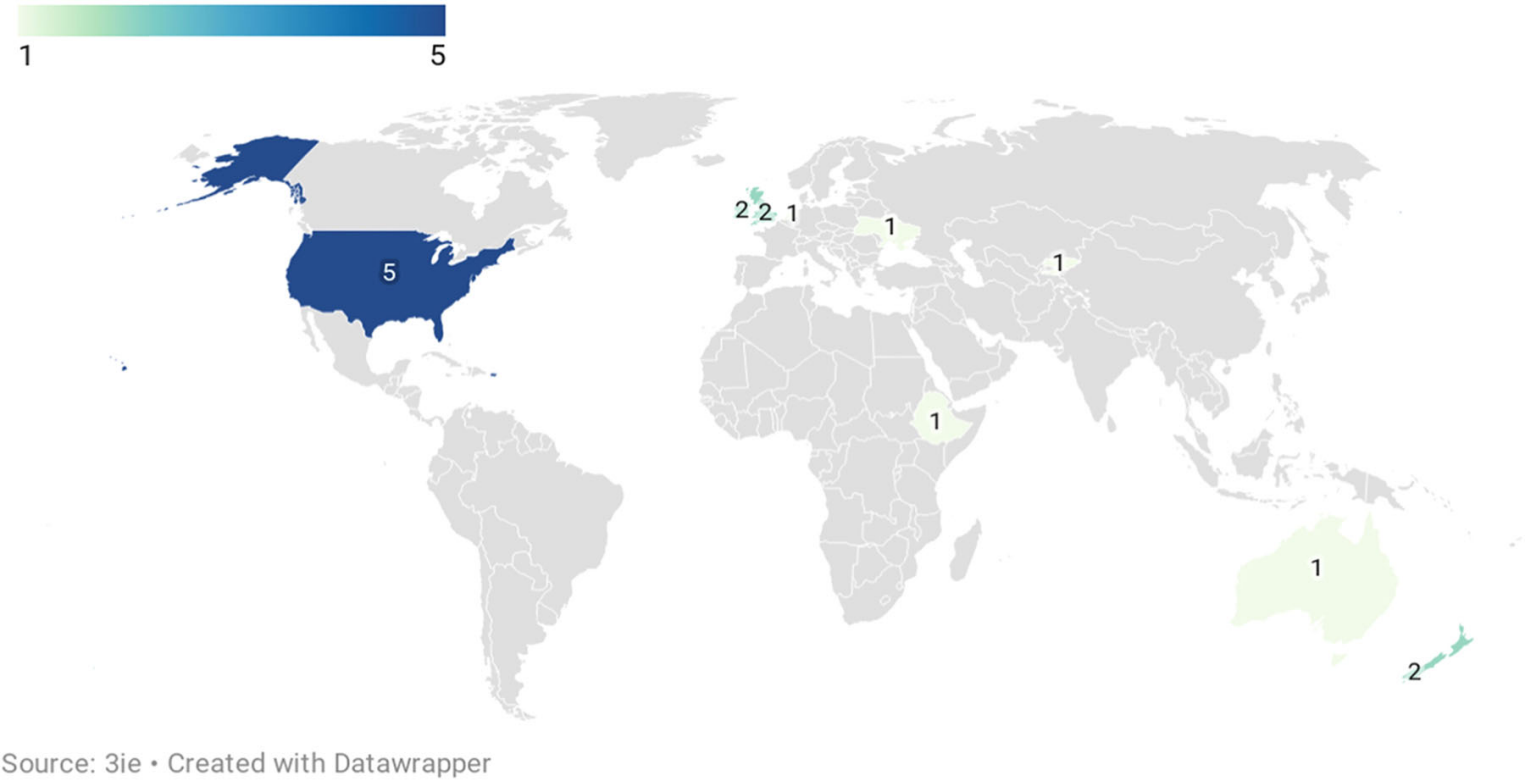
Geographic coverage of included studies

**Figure 4:**
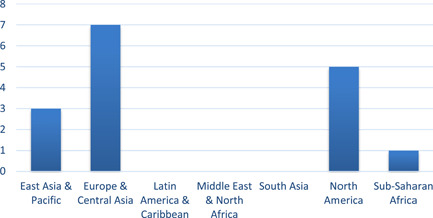
Number of included studies, by region. Regions based on World Bank classifications

**Figure 5:**
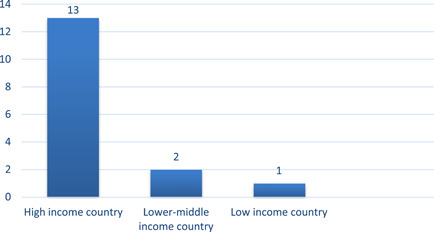
Number of included studies, by country income category. Income categories based on historical World Bank classifications for the year of publication

Over two‐thirds of the studies (69%, *n* = 11) were conducted in North America and Europe (Figure 3). Of these 11 studies, five were conducted in the United States (Alberini et al., 2016, Fowlie et al., 2018, Liang et al., 2018; Maher, 2013; Suter & Shammin, 2013), two studies in Ireland (Beagon et al., 2018, Scheer et al., 2013), two in the UK (Adan & Fuerst, 2016, Hamilton et al., 2013), one in the Netherlands (Aydin et al., 2017) and one in the Ukraine (Alberini et al., 2019). Three studies were conducted in the Pacific, respectively two in New Zealand (Grimes et al., 2016, Howden‐Chapman et al., 2007) and one in Australia (James & Ambrose, 2017). No studies were conducted in South America, with one study each conducted in Africa (Costolanski et al., 2013) and Asia (Carranza & Meeks, 2016) (Figures 4 and 5).

Thirteen of the 16 studies took place in high‐income countries (*n* = 13, 81%) using World Bank definitions (Figures 4 and 5). Costolanski et al. (2013) conducted in Ethiopia is the only included study that took place in a low‐income country and the two lower‐middle income countries are Ukraine (Alberini et al., 2019) and the Kyrgyz Republic (Carranza & Meeks, 2016).

**Figure 6:**
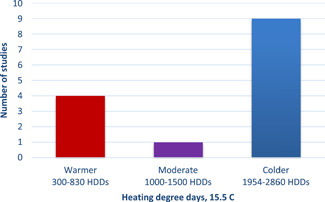
Number of studies, by climate

Most of the studies (*n* = 9) evaluated interventions in colder places (Figure 6) (1954–2860 HHDs), four studies were located in warmer locations (300–830 HHDs), and one in a moderate climate (1000–1500 HDDs). The remaining two eligible studies are not included in this graph because they examined lighting interventions (compact fluorescent lightbulbs or CFLs) which are not affected by temperature (Costolanski et al., 2013; Carranza & Meeks, 2016) (Figure 6).

#### 5.1.3 Interventions in included studies

Seven of the eligible studies examined individual EEMs, eight studies looked at bundles of two or more EEMs, and one study (Adan & Fuerst, 2016) examined both single EEMs and bundles. The most commonly examined individually EEMs were: loft/attic insulation (*n *= 4), replacement of a boiler or a heat‐pump (*n *= 4), and the installation of cavity wall insulation and efficient lighting, each examined by two studies (Table 3).

**Table 3:** Interventions of the included studies

Intervention	Studies	No. studies
Loft/attic insulation only	Adan and Fuerst (2016); Grimes et al. (2016); Hamilton et al. (2016); Maher (2013)	4
Cavity wall insulation only	Adan and Fuerst (2016); Hamilton et al. (2016)	2
Replacement boiler/heat‐pump only	Adan and Fuerst (2016); Alberini et al. (2016); Grimes et al. (2016); Hamilton et al. (2016)	4
Heating controls only	Suter and Shammin (2013)	1
Passive cooling system and design only	None	0
Energy efficient lighting only (e.g., CFL)	Carranza and Meeks (2016); Costolanski et al. (2013)	2
Window and door upgrades only	Hamilton et al. (2016)	1
District heating/cooling systems only	None	0
Information provision + one EEM only	None	0
EEM bundle (typically part of a programme)	Adan and Fuerst (2016); Alberini et al. (2019); Aydin et al. (2017); Beagon et al. (2018); Fowlie et al. (2018); Howden‐Chapman et al. (2007); James and Ambrose (2017) (includes EEM bundle plus behaviour change); Liang et al. (2018); Scheer et al. (2013)	9

In eight of the nine studies examining bundles different households received various bundles options; the specific EEMs installed were tailored for each dwelling. For instance, in the programme evaluated by Fowlie et al. (2018), an energy audit of each house determined which EEMs were appropriate and cost effective for that house. Some households replaced their furnace and installed attic and wall insulation, while others replaced their furnace and had their windows sealed, and so on.

We did not find any studies evaluating passive cooling systems, or district heating/cooling. Only one included study (James & Ambrose, 2017) looked at behavioural interventions combined with EEMs. The intervention households in this study received: a bundle of EEMs only (such as insulation, weather sealing, appliance repair and replacement, and lighting upgrades); a behavioural intervention only which included information and house operation strategies to encourage behaviour change; or both the energy efficiency and behavioural intervention (only the first and third are eligible REEIs for this review).

Some studies such as Adan and Fuerst (2016) are listed under different interventions categories because they included two or more different EEM types. For Adan et al., in particular there were four different types of EEMs, of which one was a bundle of the other three.

Additional characteristics of the 16 studies, including the 15 different programmes, are reported in Supporting Information Appendix D.

#### 5.1.4 Programme take‐up

In 10 of the 16 studies, households independently decided to install the EEMs or participate in the programme; while in the remaining six studies the households were selected by the research or programme team. Among the households that independently installed, there was perfect compliance—all the houses selected for the treatment group received the REEI and none of the comparison houses did. Among the researcher‐ or programme‐chosen households, there was perfect compliance in two studies (Beagon et al., 2018; Suter & Shammin, 2013) and in four studies there was imperfect compliance—some treatment households did not install REEIs and/or some of the comparison households did install (Carranza & Meeks, 2016; Fowlie et al., 2018; Howden‐Chapman et al., 2007; James & Ambrose, 2017). Among the imperfect compliance studies, only Howden‐Chapman et al. (2007) reported why households did not participate.

Twelve of the studies were conducted retrospectively, and used existing datasets (*n* = 7) or programme records (*n *= 6) to measure take‐up rates; two other studies conducted surveys where household self‐reported installing the EEMs (Alberini et al., 2016; Alberini et al., 2019). The final study (Costolanski et al., 2013) did not clearly describe the data source.

Most studies examining EEM bundles also reported how many households installed specific EEMs.

#### 5.1.5 Intervention funding mechanisms and context features

To better understand the context and the funding mechanisms, we conducted a search on Google in which we retrieved 18 additional documents on the programmes evaluated in the included studies.

**Figure 7:**
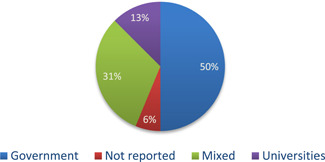
Intervention funding

**Figure 8:**
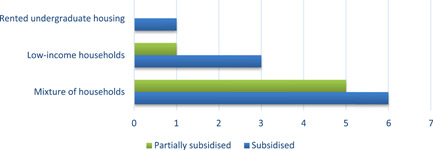
Number of studies providing subsidies, by household income

In 50% of the studies, the interventions were completely or partially subsidised by governments (*n* = 8), in 31% by a mix of public and private institutions or households (*n *= 5), 13% of the studies (*n *= 2) were funded by the research team or universities, and finally in one study the funding was not reported (Figures 7 and 8).

The government‐funded REEIs include: the SEAI Better Energy Communities scheme in Ireland (Beagon et al., 2018, Scheer et al., 2013); the Carbon Emission Reduction Target and the Community Energy Saving Programme in the UK (Adan & Fuerst, 2016); the federal Weatherization Assistance Program in the United States (Fowlie et al., 2018); the Meer Met Minder programme in the Netherlands (Aydin et al., 2017); the Warm Up New Zealand: Heat Smart scheme (Grimes et al., 2016); the Energize Phoenix programme led by the City of Phoenix, Arisona State University, and the state's largest electricity provider, Arizona Public Service (Liang et al., 2018); and the Low Income Energy Efficiency Program established by the Australian Government (James & Ambrose, 2017).

Two studies were funded by a utility company (Maher, 2013) or by a utility company (the Ethiopian Electric Power Corporation) in combination with the World Bank (Costolanski et al., 2013). In three other studies the interventions were funded through a combination of private and government funding (Alberini et al., 2016, 2019; Hamilton et al., 2016). For example, in Alberini et al. (2019), 91% (351) of the households financed the EE renovations entirely themselves, and 33 households took advantage of government programmes such as government loans and the Warm Loans programme.

Finally, for two studies (Howden‐Chapman et al., 2007; Suter & Shammin, 2013) the installed REEIs were funded by the researchers or a university.

REEI installation was subsidised regardless of household income. Four studies sampled predominantly or entirely low‐income households, 11 studies targeted samples of households at all income levels, and the remaining study involved college undergraduates. In three of the four studies with interventions targeting low‐income households, the REEI installation was fully subsidised; in the remaining study, installation was partially sub sidied (Figure 8). Six of the 11 studies that included households of any income level had fully subsidised REEIs; and REEIs were partially subsidised in five. In Alberini et al. (2019), Alberini et al. (2016) and Hamilton et al. (2016), some of the households fully funded the EE renovations themselves (Table 4) and some were subsidised. In most of the partial‐subsidy programmes, the subsidy corresponded to between 20% and 30% of the total costs. In most of the cases subsidies were provided as a reimbursement rather than an ex‐ante subsidy. In the study involving rental undergraduate housing, the REEIs were funded by the university which owned the housing.

**Table 4:** Partially subsidised programmes

Study		Partial subsidy	Programme name
Alberini et al. (2016)		Almost 56% of households received a subsidy from the government, utility, or manufacturer. The 2005 Energy Policy Act reimbursed for 10% of cost up to $500; 2009 ARRA reimbursed for 30% of cost up to $1500; starting in 2010, utilities offered between $200 and $500 for replacement heat pumps	State's EmPower rebate Programme
Alberini et al. (2019)		Roughly 91% (351) of households financed the EE renovations themselves, and 33 households used governments programmes such as government loans and the Warm Loans programme. The Warm Loans programme included reimbursement of 20% cost of boiler upgrades and 35% for other energy efficiency upgrades. The EBRD's IQ programme (only available for the last year of the period studied) provided grants up to EUR 3000, and 30,000 households in all of the Ukraine have benefitted of this programme as of September 2018	Warm Loans Programme
			European Bank for Reconstruction and Development (EBRD)'s IQ Energy
Beagon et al. (2018)		“Respond!” Housing Association received funding from the SEAI Better Energy Communities scheme for up to 50% of the cost of the project	SEAI Better Energy Communities scheme
Hamilton et al. (2016)		The data came from a database on EE installations; although the authors do not report the percentage funded by each source, some were funded by the households themselves, and others by UK government schemes (such as Warm Front, the Community Energy Savings Programme and the Carbon Emission Savings Programme	
Maher (2013)		The average rebate for attic insulation was $361 and the average rebate was $550 for central AC	Gainesville Regional Utility retrofit rebate programmes
Scheer et al. (2013)		Typically 30–35% of the installed costs of measures are grant aided	SEAI Better Energy Communities scheme

**Figure 9:**
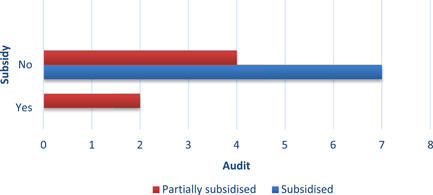
Number of studies involving audits and/or subsidies

A total of five studies (Alberini et al., 2016; Aydin et al., 2017; Fowlie et al., 2018; Liang et al., 2018; Scheer et al., 2013) reported including an audit before installation, where an expert visited the residence to assess energy usage and loss, and provided recommendations for reducing energy consumption. Among these five studies, two included a full subsidy and three included a partial subsidy (Figure 9).

#### 5.1.6 Outcomes in the included studies

To be included in the review, studies need to measure at least one of the primary outcomes (energy consumption, energy affordability, CO_2_ emissions and air quality indices and pollution levels). All 16 studies included at least one measure of energy consumption, and none of the studies measured any of the other primary outcomes. A few studies, such as James and Ambrose (2017) estimated how the intervention impacts on emissions and air pollution by multiplying the change in consumption by a factor.

Two studies also reported on secondary outcomes in the health status, comfort, and wellbeing domain: Howden‐Chapman et al., 2007 detailed indoor temperature as well as mental and physical health effects; Suter and Shammin (2013) measured monthly household ambient indoor temperature.

Three‐quarters of the included studies (*n *= 12) examined only one measure of energy consumption, either electricity, gas, or total energy (Alberini et al., 2016, 2019; Aydin et al., 2017, Beagon et al., 2018, Carranza & Meeks, 2016, Costolanski et al., 2013, Hamilton et al., 2016, James & Ambrose, 2017; Howden‐Chapman et al., 2007; Liang et al., 2017; Maher, 2013; Scheer et al., 2013, Suter & Shammin, 2013), see Table 5. Two studies reported impacts on two consumption outcomes (Adan & Fuerst, 2016; Grimes et al., 2016,) and two reported impacts on all three consumption measures (Fowlie et al., 2018; James & Ambrose, 2017).

**Table 5:** Outcomes measured by included studies

Outcome category	Outcome measure	Time period	Studies	No. of Studies
Net energy savings or consumption changes	Total energy consumption	Annual	Adan and Fuerst (2016); Howden‐Chapman et al. (2007)	2
		Monthly	Fowlie et al. (2018); Grimes et al. (2016)	2
		Daily	James and Ambrose (2017)	1
	Electricity consumption	Annual	‐	0
		Monthly	Alberini et al. (2016; Carranza and Meeks (2016); Costolanski et al. (2013); Fowlie et al. (2018); Grimes et al. (2016); Liang et al. (2017); Maher (2013)	7
		Daily	James and Ambrose (2017)	1
	Gas consumption	Annual	Adan and Fuerst (2016); Aydin et al. (2017); Beagon et al. (2018); Hamilton et al. (2013); James and Ambrose (2017); Scheer et al. (2013)	6
		Quadrimester	Alberini et al. (2019)	1
		Monthly	Fowlie et al. (2018); Suter and Shammin (2013)	2
Health status, comfort, and wellbeing			Howden‐Chapman et al. (2007); Suter and Shammin (2013)	2

No studies reported the following outcomes which were specified in the protocol: energy security, air quality index, income savings, GHG emissions, job creation, building stock value.

The measurement units for total energy and electricity consumption are typically reported in kWh except for Fowlie et al. (2018) who reported consumption in MMBtu. Gas consumption is commonly measured in m^3^ (Alberini et al., 2019; Aydin et al., 2017), or kWh (Adan & Fuerst, 2016; Beagon et al., 2018; Hamilton et al., 2016; Scheer et al., 2013) or Ccf (Suter & Shammin, 2013) or MMBtu (Fowlie et al.).

The energy consumption data was obtained from utility companies, gas and electricity meter operators, or in one study, household energy bills (Alberini et al., 2019). To measure indoor temperature and humidity, one study installed sensors (Suter & Shammin, 2013) and a second installed “data‐loggers” (Howden‐Chapman et al., 2007). Howden‐Chapman et al. (2007) measured health status using interviewer‐administered or self‐administrated surveys with the residents or health care providers. Many of these health measures are subsets of or adopted from existing health measurement scales, such as SF‐36 scales (Howden‐Chapman et al., 2007).

### 5.2 Risk of bias in included studies

When different studies estimate different impacts for the same REEI, we suggest focusing on the impacts estimated by low risk of bias studies. Risk of bias assesses the likelihood that something other than the intervention caused any change in energy consumption. For example, a study with different treatment and comparison groups—such as treatment households being more environmentally conscious—would have a high risk of bias because those group differences are likely to also cause differences in energy consumption. Thus, focusing on low risk of bias studies provides the most reliable evidence of how REEIs affect energy consumption.

High risk of bias studies can provide initial information when there are no low risk of bias studies examining an REEI in a particular context or with a specific population. In those situations, high risk of bias studies provide useful preliminary evidence, because all included studies, regardless of risk of bias, have a rigorous design and thus meet a minimum level of quality.

#### 5.2.1 Risk of bias summary

**Figure 10:**
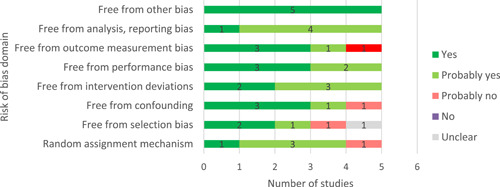
Risk of bias for included studies using randomised designs

Of the 16 eligible studies, five were appraised as having a low overall risk of bias (“probably yes” or “yes” in the eight risk of bias domains, see Figure 10 for domains), two studies were appraised as *some concerns* due to incomplete reporting, and the other nine studies were appraised as having a high overall risk of bias (rated “no” or “probably no” in at least one domain). We appraised risk of bias using slightly different criteria for randomised trials and quasi‐experimental designs.

Three of the five randomised trials were rated as low overall risk of bias (see Figure 10 and Supporting Information Appendix Table E1 for the appraisal of each study on each criterion), and two of the 11 quasi‐experiments were rated as low overall risk of bias (see Supporting Information Appendix Table E2). One randomised trial (Carranza & Meeks, 2016) and one quasi‐experiment (Maher, 2013) were rated as *some concerns* because the study did not report information needed for the appraisal and the author did not respond to a request for additional information.

Three of the five included randomised trials were appraised as having overall low risk of bias (Fowlie et al., 2018, Howden‐Chapman et al., 2007; Suter & Shammin, 2013). For the one study with *unclear* appraisal on selection bias (Carranza & Meeks, 2016), the authors did not report how 14 assigned but unsurveyed clusters were chosen (i.e., whether this attrition was random); otherwise, there were no serious concerns with this study. One randomised trial (James & Ambrose, 2017) was appraised with concerns in four domains: compromised random assignment (several households were assigned based on researcher perceptions of responsiveness); high attrition likely related to whether the household was assigned to treatment or comparison group; important baseline differences between groups; and the authors were more likely to have outcome data from control households for certain months. For the other studies, there was less risk of performance bias, outcome measurement bias, or analysis bias because outcomes were typically from administrative data.

**Figure 11:**
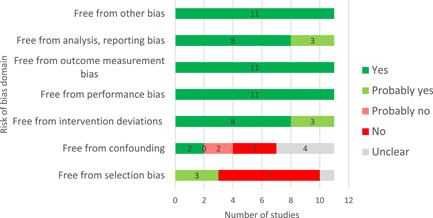
Risk of bias for included studies using quasi‐experimental designs

Two of the quasi‐experiments were appraised as having a low overall risk of bias (Adan & Fuerst, 2016; Grimes et al., 2016). The most common issues for the included quasi‐experiments were selection bias and confounding, with only three of the 11 studies being appraised as low risk of bias in both those domains (Figure 11). The three studies with unclear appraisals on the confounding domain (Alberini et al., 2016; Hamilton et al., 2016; Maher, 2013) did not report the statistics needed to assess baseline equivalence. Similar to the randomised trials, the quasi‐experimental outcomes were typically administrative records from utility companies, so there was less risk of bias in the other domains.

### 5.3 Quantitative synthesis of results

#### 5.3.1 Presentation of results

We report the magnitude of energy consumption impacts in two ways: (1) standardised mean difference (Hedges' *g*), and (2) change in kilowatt hours (kWh). Hedges' *g* enables impacts to be compared across studies/interventions and is thus our primary reporting metric, used in the text and forest plots. However, because Hedges' g impacts are in SD units, a nonintuitive metric, we also report impacts in kWh. Hedges' *g* and average difference in temperature or health are also reported for other outcomes.

To facilitate understanding of impacts reported as Hedges' *g*, we provide intuitive benchmarks estimated in other studies. First, one recent study (Huebner et al., 2015) estimates that adding one additional member to a household was associated with an increase in household energy consumption by 0.46 SDs (Hedges' *g* = 0.46). A second study (Huebner et al., 2016) estimated that houses with an electric clothes dryer consumed 0.22 SDs of energy more than those who hung their clothes to dry (although these estimates were not calculated through a counterfactual analysis that can establish causation, these studies do control statistically for important factors, such as amount of livable space.)

We estimate average impacts using random effect meta‐analysis because we seek to make broader inferences and relevant factors varied across studies. We present heterogeneity statistics (*Q* statistic, *τ*, and *I* ^2^) in Supporting Information Appendix F, along with prediction intervals in the forest plots. Random‐effects meta‐analysis can provide unreliable estimates for the between‐studies variance (*τ* ^2^) when the analysis includes a small number of studies, as in this review. As a sensitivity check, Supporting Information Appendix G presents overall averages estimated using a fixed‐effects meta‐analysis. Because of the small number of studies and the diverse interventions, we do not conduct a meta‐regression to systematically explore sources of heterogeneity. Instead, we describe possible explanatory factors in the text.

#### 5.3.2 Forest plots

**Figure 12:**
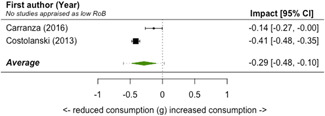
Impact of highly subsidised compact fluorescent light bulbs. For individual studies, the rightmost column and the horizontal lines indicate the 95% confidence intervals (we can be 95% confident that this interval captures the actual impact). For the average impacts, the confidence interval is displayed in the rightmost column and represented by the width of the diamond, while the dashed horizontal lines indicate the prediction interval (the range in which the future impact will likely fall)

When there are two or more studies, results are presented in forest plots (such as Figure 12, a common graphic that presents estimated impact for each study, variation between studies, and average impact across studies.

**Figure 13:**
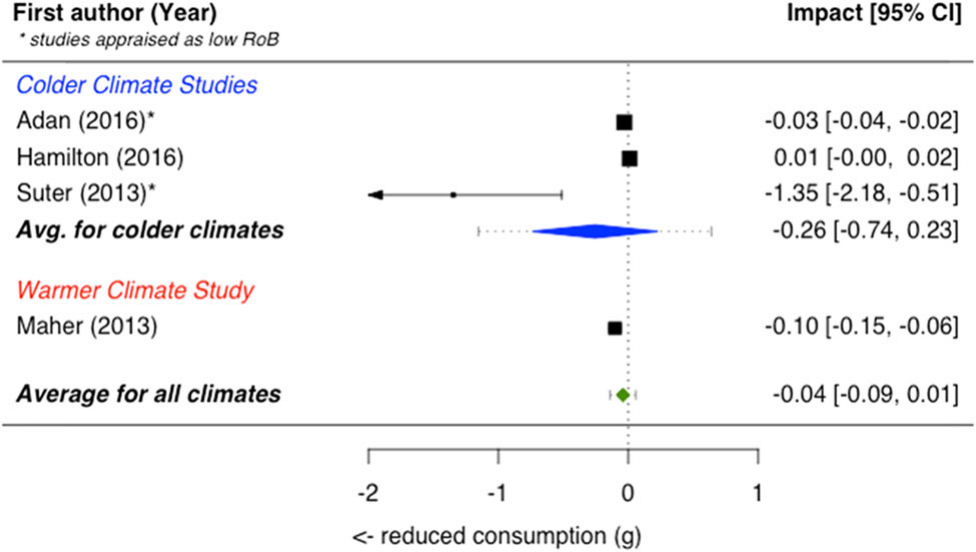
Impacts of attic or loft insulation only, by climate subgroup. Because of the large impact estimated in Suter et al., the scale extends to −2 rather than −1 as in the other forest plots. For individual studies, the rightmost column and the horizontal lines indicate the 95% confidence intervals (95% of the time this interval will capture the actual impact). For the average impacts, the confidence interval is displayed in the rightmost column and represented by the width of the diamond, while the dashed horizontal lines indicate the prediction interval (the range in which the impact will fall for 95% of the population)

Studies are first categorised by climate subgroup for the heating/cooling REEIs, and each study is presented in a separate row (such as Figure 13). When there are multiple studies in a given climate, the last row for the climate presents the average impact for the climate subgroup. The final row in the plot presents the average for all studies across all climates.

The first column in the plot lists the study's first author and publication year. The second column graphically presents the study's Hedges' *g* on a scale from −1 to +1, with a dotted vertical line indicating 0 Hedges' *g* (no impact). The point estimate for each study is represented with a box and the size of the box reflects the study sample size. The point estimate is the best estimate for the actual impact, but there is variation due to the randomness of the assignment/sampling process; we report this uncertainty using CIs and predictions intervals. The 95% CIs for each study are indicated with horizontal lines—95% of the time this interval will capture the actual impact. The average impact across studies is displayed with a green diamond and the width of the diamond indicates the CI—95% of the time this interval will capture the actual average impact. Finally, the prediction intervals for the average impact are indicated with horizonal dashed lines—if we predict the REEI impact for a randomly selected population, there is 95% chance the impact will fall in this interval (Borenstein, 2019). The third column in the plot numerically reports the impact and 95% CIs.

#### 5.3.3 Results from one study not presented

One eligible study (James & Ambrose, 2017) did not report sufficient information to calculate an effect size and the lead author did not respond to a request for this information. Accordingly, findings from this study are not reported in this section (this was the one study that occurred in a moderate climate).

#### 5.3.4 Supplemental findings

Because some of the studies examined different interventions using the same comparison group, creating dependent comparisons, these supplemental impacts are reported in Supporting Information Appendix H.

#### 5.3.5 Source of data

All the data used for the following analysis were obtained from the published studies included in this review.

#### 5.3.6 Lighting interventions

Two included studies examined providing households with up to four CFLs. Each study estimated that providing CFLs to households significantly reduced electricity consumption, with the average impact equal to Hedges' *g* = −0.29. The Costolanski et al. (2013) intervention allowed Ethiopian households to exchange incandescent bulbs for CFLs, while the Carranza and Meeks (2016) intervention enabled Kyrgyz households to buy CFLs at highly subsidised prices.

Impacts varied between the studies, with Carranza and Meeks (2016) estimated impact roughly one‐third of the impact estimated by Constolanski et al. (2013). One possible reason for the lower impact in the Carranza and Meeks study was somewhat lower participation (households received 3.2 CFLs on average and 12% of households received no CFLs compared to 98% of households in Costolanski et al. receiving four CFLs). Moreover, although neither study was appraised as low risk of bias, selection bias and confounding were much larger risks to the Costolanski et al. findings (see Supporting Information Appendix E). However, the meta‐analysis weights the larger sample Costolanski et al. study (3998 households included in the analysis) more when computing the average impact than the Carranza and Meeks study (899 households) (Table 6).

**Table 6:** Study‐reported impacts of compact fluorescent light bulbs (kWh)

Study	Annual impact per household in kWh	Hedges' g
Carranza and Meeks (2016)	−301	−0.14
Costolanski et al. (2013)	−424	−0.41

#### 5.3.7 Heating and cooling interventions

Because climate directly determines how much heating or cooling is needed, we synthesise impacts for heating and cooling interventions separately by climate as determined by annual HDD—specifically, the colder and warmer climate subgroups as reported in Figure 6—as well as an average across all studies and climates. In the forest plots, the meta‐analytic average for colder climates is reported using a blue diamond, for warmer climates using a red diamond, and an overall average impact across climates using a green diamond (when there is only one study in a climate, we do not report a meta‐analytic average for that climate). Presenting studies separately by climate does not mean that any differences are due to climate; differences between climates might be due to other factors.

#### 5.3.8 Attic insulation without any other EEMs

Four studies examined the impact of attic insulation on energy consumption. Three studies took place in colder climates with gas consumption as an outcome (Adan & Fuerst, 2016; Hamilton et al., 2016; Suter & Shammin, 2013), and one study took place in a warmer climate, with the electricity used to run air conditioning consumption as the outcome (Maher, 2013). The average impact across all climates was statistically insignificant (Hedges' *g* = 0.04).

Two of the three studies examining attic insulation in cold climates estimated impacts less than Hedges' *g* = 0.05, while the third study estimated a much larger impact; the average impact in colder climates was not statistically significant (see Figure 13). Adan and Fuerst (2016), a low risk of bias study, found a smaller reduction in energy consumption (Hedges' *g* = −0.03), although the sample was so large (over 150,000 households) that this impact was statistically significant. Hamilton et al. (2016) also estimated a smaller impact for a large sample of roughly 105,000 households (Hedges' *g* = 0.01). However, Suter and Shammin (2013) another low risk of bias study, found a larger impact on reducing energy consumption (Hedges' *g* = –1.35), so large that the forest plot scale is different.

The descriptions of attic insulation are incomplete, but Suter et al. might have installed thicker attic insulation than households in Hamilton et al. and Adan et al. Specifically, Hamilton et al. noted that most households installed insulation between 5 and 75 mm thick. Although Adan et al. do not report the amount of insulation, like Hamilton et al., the study took place in the UK and involved some of the same energy efficiency programmes and might have also installed similarly thin installation. Limited additional insulation would not be expected to change consumption much. In contrast, the Suter et al. homes were roughly 100 years old, “with minimal amounts of prior insulation… and with occupants that typically heat their homes to approximately 72 degrees F” (p. 559), and the attic insulation cost roughly $1000 indicating a significant amount of insulation was installed.

There are other possible explanations for the high Suter et al. finding. Suter et al. note that the sample houses were “relatively homogeneous in their size and characteristics” (p. 554); this similarity will lead to less variation in consumption between houses (i.e., a smaller SD) and a larger Hedges' *g*. Table 7 indicates that although the Suter et al. impact was large, in absolute terms it was less than twice that of Maher. Moreover, as the small box in the plot indicates, this study involved only 24 households and so the impact is not precisely estimated (because the random‐effects meta‐analysis weights by precision, this impact does not have much effect on the average impact). Finally, the houses in Suter et al. were rentals that included utilities with the rent, and these dwellings typically use more energy (Leth‐Petersen & Togeby, 2001). Thus, households in Suter et al. might have been using more energy at baseline enabling the insulation to have a larger impact.

**Table 7:** Study‐reported impacts of attic insulation (kWh)

Study	Annual impact per household in kWh (percentage change in consumption)	Hedges' *g*
Adan and Fuerst (2016)*	(−3 percentage points annually)	−0.03
Hamilton et al. (2016)	+153	0.01
Suter and Shammin (2016)*	−548	−1.35
Maher (2013)	−299	−0.10

*Notes*: Although Adan and Fuerst (2016) do not report using the natural log of energy consumption as an outcome, their description of impacts implies that the estimation used the natural log of energy consumption (such as loft insulation leads to “an estimated reduction in gas consumption of 3.1%”; p. 1213).

* Indicates low risk of bias.

A fourth study (Maher, 2013) examined the impact of attic insulation in a warmer climate (7526 households) and found that insulation significantly reduced electricity consumption. This study occurred in a hot, humid area (Gainesville, Florida, United States), a location with high need for air conditioning.

#### 5.3.9 Electric heat pumps without any other EEMs

Two studies examined the impact of installing electric heat pumps. The average impact across studies was ‐0.11 and not statistically significant. However, there was substantial variation between the two studies. Replacing older pumps with more efficient versions significantly reduced electricity consumption (335 households) in a colder climate (Alberini et al., 2016), but Grimes et al. found a smaller, significant increase in energy consumption from installing new heat pumps (24,164 households). The authors note that part of the “increased energy use occurred at warmer temperatures, when heat pumps were likely used as air conditioners” (p. 165). This represents new energy consumption because traditionally New Zealand houses did not have air conditioning (French et al., 2009). Thus, in contexts where older heat pumps are being replaced by more efficient models, the Alberini et al. estimates are likely more relevant.

Although Grimes et al. was appraised as low risk of bias, the study results do not fully capture heat pumps' impact on total energy consumption. Specifically, the authors note that they did not measure solid fuel consumption, such as wood and coal stoves. Yet solid fuel was used to generate roughly 56% of home heating energy in New Zealand at the time of the study (French et al., 2009). Thus, installing heat pumps to replace wood or coal stoves would increase measured electricity consumption, but also reduce consumption of unmeasured solid fuels.

**Figure 14:**
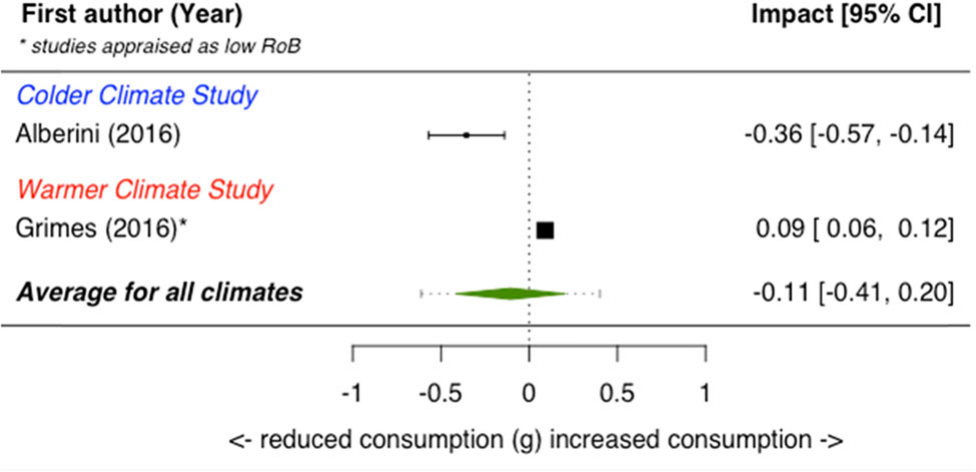
Impacts of electric heat pump only, by climate subgroup. For individual studies, the rightmost column and the horisontal lines indicate the 95% confidence intervals (95% of the time this interval will capture the actual impact). For the average impacts, the confidence interval is displayed in the rightmost column and represented by the width of the diamond, while the dashed horisontal lines indicate the prediction interval (the range in which the impact will fall for 95% of the population)

An earlier version of the paper (Grimes et al., 2011) reports two supplemental analyses consistent with the possibility that the installed heat pumps reduced total energy consumption. In the first analysis, the authors estimated impacts among subgroups of households that did and did not use unmeasured fuels before installing heat pumps. While households using unmeasured energy sources before installation increased measured energy (electricity + piped gas) consumption after installation, households that used measured sources preinstallation typically reduced total? energy consumption at colder outside temperatures after installation. Although these differences were not statistically significant, the authors note the analysis was underpowered as they did not have data for most households' preinstallation energy sources. In a second subsample analysis, comparing households that did and did not have access to piped gas before installation, the authors find that households with gas—who presumably replaced gas heaters with heat pumps—significantly reduced energy (gas + electricity) consumption at colder temperatures while households without gas—presumably more likely to use unmeasured energy sources—increased total measured energy consumption at colder temperatures (as only 13.6% of sample households had access to gas, this subsample had a small effect on overall impacts) (Figure 14 and Tables 8, 9, 10).

**Table 8:** Study‐reported impacts of electric heat pumps (KWh)

Study	Annual impact per household in KWh (percentage change in consumption)	Hedges' *g*
Alberini et al. (2016)	(−8 percentage points monthly)	−0.36
Grimes et al. (2016)*	+140	0.09

* Indicates low risk of bias.

**Table 9:** Study‐reported impacts of individual EEMs (kWh)

Study	Annual impact per household in kWh (percentage change in consumption)	Hedges' *g* (SE)
Boiler replacement		
Adan and Fuerst (2016)*	(−4 percentage points annually)	−0.04 (0.004)
Cavity wall insulation		
Adan and Fuerst (2016)*	(−10 percentage points annually)	−0.11 (0.007)
Efficient central air conditioning replacement		
Maher (2013)	−1395	−0.51 (0.023)

* Indicates low risk of bias.

**Table 10:** Study‐reported impacts of EEM bundles that vary by household (KWh)

Study	Annual impact per household in KWh (percentage change in consumption)	Hedges' *g*
Alberini et al. (2019)	(−2 percentage points quadrimesterly)	−0.08
Aydin et al. (2017)	−4382	−0.64
Beagon et al. (2018)	−1277	−0.36
Fowlie et al. (2018)*	(−19 percentage points monthly)	−0.12
Scheer et al. (2013)	−3663	−0.57
Howden‐Chapman et al. (2007)*	(−10 percentage points annually)	−0.29
Liang et al. (2018)	(−10 percentage points monthly)	−0.35

*Notes*: Although Howden‐Chapman et al. (2007) do not report using the natural log of energy consumption as the outcome, their description of impacts implies the outcome was the natural log of energy consumption (such as treatment “households consuming 92% of that consumed by control households” (p. 4) and reporting of geometric means).

* Indicates low risk of bias.

#### 5.3.10 Other individual EEMs

One included study examined the impact of installing a more efficient boiler or installing cavity wall insulation (Adan & Fuerst, 2016). The samples in these analyses were independent (i.e., did not share a comparison group) and involved roughly 360,000 households (boiler) and 103,533 households (cavity wall). Another study, involving 7526 households, examined the impact of replacing central air conditioning with a more efficient system (Maher, 2013).

Adan et al. find that the impact of cavity wall insulation was roughly three times larger in magnitude than their estimated impact for loft insulation; the authors do not explain why wall insulation was more impactful. Maher finds a large impact of replacing central air conditioning, likely caused by two features: (1) the study was conducted in Gainesville, Florida, a hot and humid climate with a strong need for air conditioning, and (2) the subsidy programme required that the replacement air conditioning system be rated highly efficient by the US Environmental Protection Agency.

#### 5.3.11 EEM bundles

The previous sections have synthesised and/or reported the impact of installing one EEM, but households often install more than one EEM at a time. Eight studies examined the impact of these so‐called bundles of multiple EEMs, and we classify these interventions into two categories:

Bundles where the EEMs installed in each household varied because each household chose which EEMs to install (i.e., households within each study received different bundles).

Bundles where each household installed the same bundle (one study (Adan & Fuerst, 2016) involving four independent comparisons).

Five of the seven studies in the first category examine bundles installed after an energy audit; for these bundles, the specific EEMs installed in each household varied based on the audit (i.e., households often installed tailored interventions based on dwelling needs assessed by a professional). A supplemental analysis for these studies is reported in Supporting Information Appendix I.

Each category is relevant for a different research question, and accordingly we analyze each category separately.

#### 5.3.12 EEM bundles, where EEMs installed in each household vary

**Figure 15:**
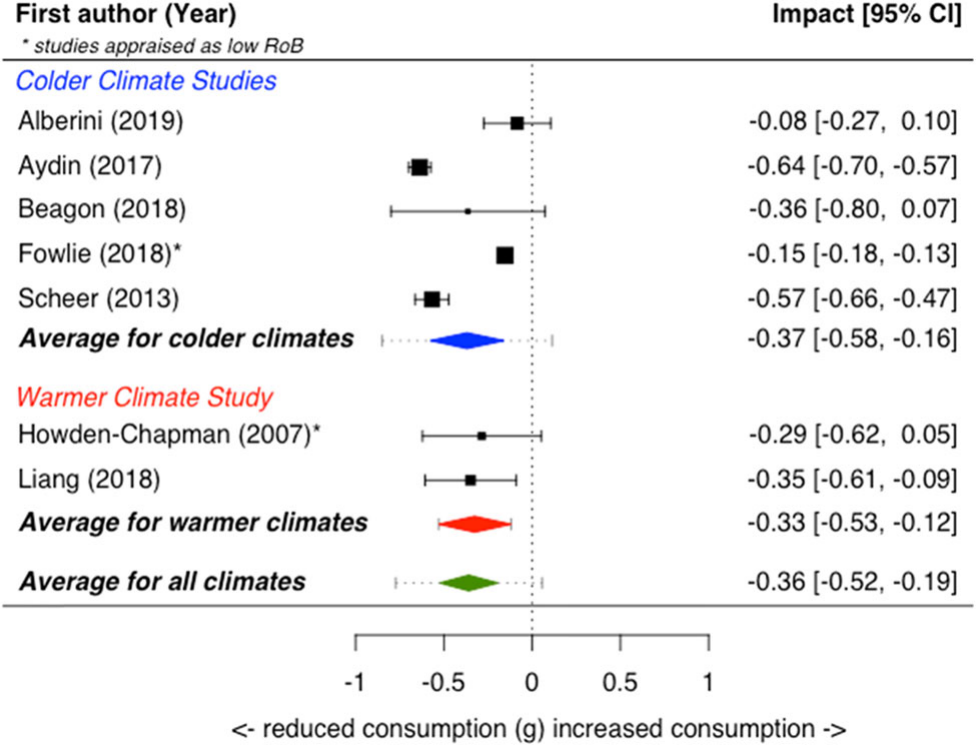
Impacts of EEM bundles that vary by household, by climate. For individual studies, the rightmost column and the horizontal lines indicate the 95% confidence intervals (95% of the time this interval will capture the actual impact). For the average impacts, the confidence interval is displayed in the rightmost column and represented by the width of the diamond, while the dashed horizontal lines indicate the prediction interval (the range in which the impact will fall for 95% of the population)

The included studies uniformly found that installing bundles reduced residential energy consumption (Figure 15), with an average impact of 0.36 SD units that was statistically significant. Although the bundles and contexts were diverse, most estimated reductions in energy between −0.29 and −0.64. The two exceptions were Fowlie et al. (2018) (−0.15 and statistically significant) and Alberini et al. (2019) (−0.08 and not statistically significant). The number of households included in the analysis ranged from 45 (Beagon et al., 2018), 136 (Howden‐Chapman), 231 (Liang et al., 2018), 429 (Alberini et al., 2019), 5198 (Aydin et al., 2017), roughly 25,000 (Fowlie et al., 2018) to over 150,000 (Scheer et al., 2013).

Overall, the two low risk of bias studies indicate an impact of between −0.15 to −0.29. Unlike the other studies that estimated an average treatment‐on‐treated effect, the Fowlie et al. estimates are for *compliers, those participants who complied with their assignment*, roughly six percent of the sample (the intervention encouraged and helped eligible families to apply to the REEI programme). In addition, the Fowlie et al. effect size calculations relied on additional assumptions (see Section 4.3.5) that might have affected the estimated effect size.

The Alberini et al. estimates are lower than others, but most study households only installed one or two EEMs, typically wall insulation by itself or with new windows (the authors note that instances where a sample “household does more than one efficiency upgrades are not numerous”; p. 22). Given the limited nature of the intervention bundle, the impact of the intervention (‐0.08) is similar to the impact of wall insulation only estimated by Adan and Fuerst (2016) (−0.11).

#### 5.3.13 All bundles: Identical EEMs installed in each household

Unlike the previous studies that examine bundles where the EEMs differed by residence, using a retrospective analysis, Adan and Fuerst (2016) examined the impact of identical bundles installed in each residence. Specifically, they study all four possible combinations of attic insulation, cavity wall insulation, and boiler replacement (see Table 11).

**Table 11:** Study‐reported impacts of identical bundles (KWh)

Study	Percentage change in consumption	Hedges' *g* (SE)
Attic and wall insulation		
Adan and Fuerst (2016)*	(−7 percentage points annually)	−0.06 (0.006)
Attic insulation and boiler		
Adan and Fuerst (2016)*	(−5 percentage points annually)	−0.04 (0.012)
Wall insulation and boiler		
Adan and Fuerst (2016)*	(−13 percentage points annually)	−0.11 (0.017)
Attic and wall insulation, and boiler		
Adan and Fuerst (2016)*	(−10 percentage points annually)	−0.09 (0.019)

*Note*: The percentage change for Adan and Fuerst (2016) is annual.

* Indicates low risk of bias.

The authors estimate that each bundle reduced gas consumption, although the effects were always less than Hedges' *h* = −0.11. All of the samples have more than 10,000 households, with precisely estimated impacts, thus even when the reduction in consumption is small it is statistically significant.

The impact of installing additional EEMs in the bundles does not appear to be additive. For example, the impact of installing wall insulation only was −0.11 (see Table 11) and the impact of installing a boiler only was −0.04, but the impact of installing a boiler and wall insulation was −0.11, not 0.15. Similarly, the largest reduction in Table 11 occurred when households installed both cavity wall insulation and a boiler, and this reduction was larger than when the household installed cavity wall insulation, a boiler, and loft insulation. The authors label this pattern “less straightforward” and believe the lack of additivity is due to the prebound effect—households in the least efficient residences consume the least energy (prebound effect) and were most likely to install multiple EEMs; thus, households that consume less energy through energy behaviour were more likely to install bundles but their behaviour meant that they were also least likely to benefit from bundles.

#### 5.3.14 Indoor temperature and health outcomes

Few studies that estimated REEI impacts on energy consumption also examined other outcomes. Two included studies, examining different REEIs, reported indoor temperature outcomes (Howden‐Chapman et al., 2007; Suter & Shammin, 2013). (In addition to the randomised experiment included in this synthesis, Fowlie et al., 2018 also used a quasi‐experimental design to estimate impacts on indoor temperature outcomes; however, this design was ineligible.) One study reported impacts on several self‐reported health outcomes (Howden‐Chapman et al., 2007).

#### 5.3.15 Indoor temperature

Both studies estimated that households installing an REEI slightly increased indoor temperatures during the winter, although the impact was only statistically significant for Howden‐Chapman et al. (Hedges' *g* = 0.35). These increases are consistent with a small rebound effect. Howden‐Chapman recorded main bedroom temperatures during the winter in 140 randomly selected houses. Suter et al. installed temperature and humidity sensors in each home, recorded temperature every 10 min (the data were aggregated by month) and focused on indoor temperatures during winter months (November through to March). Smaller impacts in Suter et al. are expected—households did not pay for utilities, and thus had little incentive to keep their house at a lower temperature to save money (Table 12).

**Table 12:** Study‐reported impacts on indoor temperature in winter (°C)

Study	Change in temperature (°C)	Hedges' g (SE)
EEM bundle that varies by household		
Howden‐Chapman et al. (2007)*	+0.50	0.35 (0.17)
Attic insulation		
Suter and Shammin (2013)*	+0.41	0.23 (0.40)

*Note*: Suter et al. impacts were reported in Fahrenheit and converted to Celsius by multiplying by 5/9.

* Indicates low risk of bias.

#### 5.3.16 Health outcomes

Of the included studies, only Howden‐Chapman et al. (2007) examined health outcomes, and this study included outcomes in the following domains: self‐reported mould (allergen) in the house, self‐reported low vitality, self‐reported low happiness, self‐reported fair or poor health, self‐reported winter colds or flu, self‐reported wheezing, and self‐reported phlegm. We do not include health care usage outcomes (such as visits to general practitioners) in this SR because the desired direction is unclear (e.g., a visit to a primary care doctor could be either a good or bad outcome).

For each outcome, Howden‐Chapman et al. found that installing the EEM bundle improved self‐reported health (Table 13) and the impacts were statistically significant.

**Table 13:** Study‐reported impacts on health outcomes

Study	Change in temperature (°C)	Hedges' *g* (SE)
Self‐reported any observed mould		
Howden‐Chapman et al. (2007)	−30 percentage points	−0.79 (0.07)
Self‐reported low vitality (% reporting bottom three categories)		
Howden‐Chapman et al. (2007)	−13 percentage points	−0.37 (0.05)
Self‐reported low happiness (% reporting bottom half of scale)		
Howden‐Chapman et al. (2007)	−5 percentage points	−0.32 (0.08)
Self‐reported fair or poor health		
Howden‐Chapman et al. (2007)	−8 percentage points	−0.38 (0.07)
Self‐reported winter colds or flu		
Howden‐Chapman et al. (2007)	−13 percentage points	−0.34 (0.04)

*Note*: To be consistent with Hedges' *g*, percentages are postintervention treatment—comparison unadjusted. The baseline differences for the analytic samples were less than two percentage points.

### 5.4 Subgroup analysis

#### 5.4.1 Household socioeconomic status

**Figure 16:**
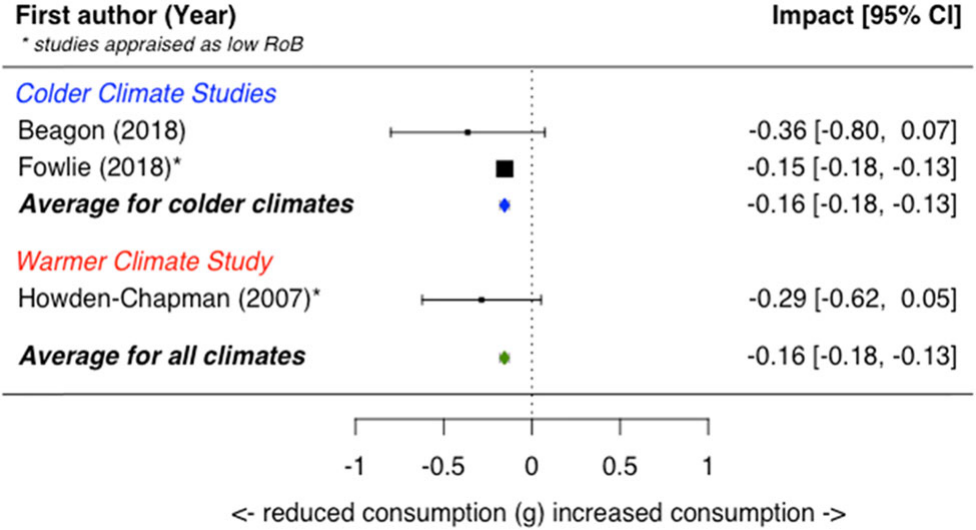
Impacts of EEM bundles for low‐income households only, by climate. For this analysis, low‐income refers to relative income status within a country, not based on absolute income level. The confidence interval for the overall average, displayed in the rightmost column and the width of the diamond, will include the actual population mean 95% of the time. The narrow prediction interval, the bars around the average for all climates, indicate the interval in which 95% of the population will have an impact. EEM;

Two studies examined interventions implemented with only low‐income households (Irish social housing in Beagon 2018; US Weatherization Assistance Program in Fowlie et. al. 2018) and one study included households that mostly lived in lower‐income areas (Howden‐Chapman et al., 2007). The intervention in each of these studies was an EEM bundle, and all of the studies estimated that bundles reduced energy consumption (see Figure 16).

The small number of studies and the much larger sample size in one study limit the generalisability of the subgroup analysis. Specifically, because the meta‐analysis weights studies by precision, when rounded, the average impact for low‐income household studies (0.16) is almost the same as the impact estimated by Fowlie et al., a study with 28,790 households (the other two studies have 181 households in total). Two of the three studies (Fowlie et al. and Howden‐Chapman et al.) were appraised as low risk of bias. Given the small number of studies involving low‐income households, further investigation of differences between these households and other households was not possible.

#### 5.4.2 Region of residency (European Union‐27 and the UK)

**Figure 17:**
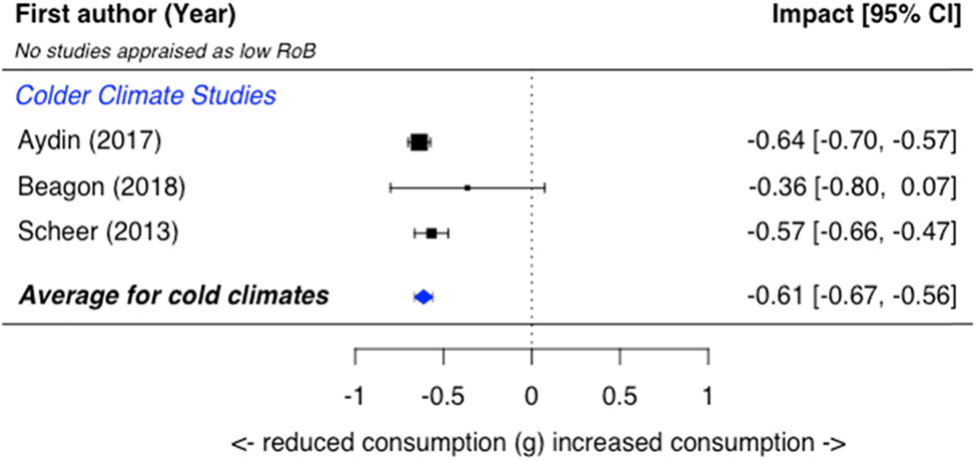
Impacts of EEM bundles for EU households only, by climate. EEM; EU;

Five studies examined interventions conducted in EU‐27 or the UK: two in Ireland (Beagon et al., 2018; Scheer et al., 2013), one in the Netherlands (Aydin et al., 2017) and two in the UK (Adan & Fuerst, 2016; Hamilton et al., 2016). The studies that occurred in Ireland and the Netherlands examined a similar intervention—bundles that vary by household—and are synthesised (Figure 17).

All of these studies took place in colder climates, and the average impact is large (0.61) and precisely estimated, but none of these studies was appraised as low risk of bias. Because the meta‐analysis weights studies by precision, the Beagon et al. impact, based on 45 households is not heavily weighted.

### 5.5 Sensitivity analysis

Neither of the sensitivity analyses led to contradictory findings, although the smaller sample sizes led to unreliable estimation.

#### 5.5.1 Remove individual studies

For the two meta‐analyses with three or more studies (attic insulation and EEM bundles that vary by household), we assessed whether the results of the meta‐analysis were sensitive to the removal of any single study (see Supporting Information Appendix J). This analysis found minor differences for the meta‐analysis of bundle interventions and always in the expected direction (i.e., removing studies with impacts below the average increased the average and vice versa). For the attic insulation meta‐analysis, removal of Adan and Fuerst (2016), Hamilton et al. (2016), and Maher (2013) led to unreliable estimation with accordingly large standard errors.

#### 5.5.2 Remove high risk of bias

When limiting syntheses to those studies appraised as low risk of bias (*n* = 5), only one meta‐analysis is possible—EEM bundles that vary by household. This meta‐analysis included two studies: Fowlie et al. (2018) and Howden‐Chapman et al. (2007).

**Figure 18:**
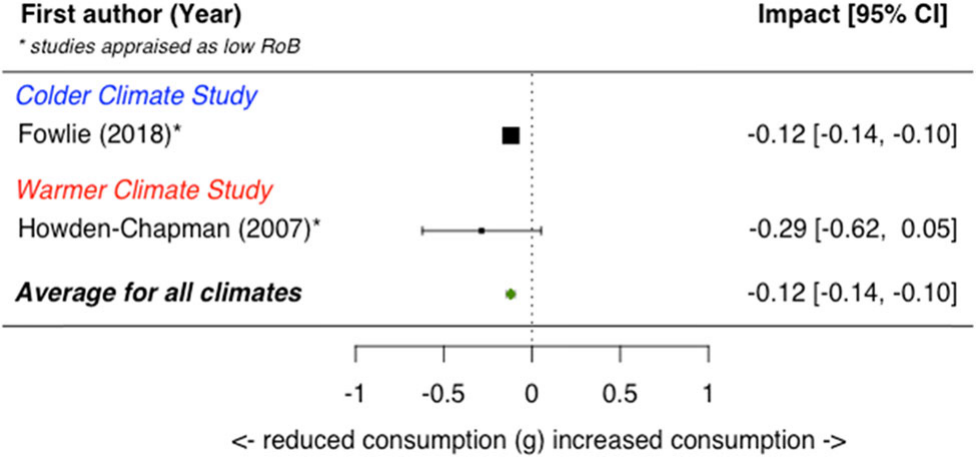
Impacts of EEM bundles for low risk of bias studies only, by climate. EEM;

The average effect size is −0.16 and statistically significant across the two low risk of bias studies is similar to the Fowlie et al. estimate of −0.15 (see Figure 18); the Fowlie et al. estimate dominates the meta‐analysis because it is much more precisely estimated as the study has a sample size more than 200 times larger than Howden‐Chapman et al.

#### 5.5.3 Cost analyses

Eight studies also conducted some type of cost analysis, estimating the cost of saving energy, reducing pollution, or calculating the rate of return on investment (Table 14). These calculations are based on study estimates of how much REEIs affect energy consumption and other assumptions. Thus the larger the estimated reduction, holding other assumptions constant, the lower the cost estimate or the higher the rate of return.

**Table 14:** Include studies' cost analyses, by REEI type

Study	Type of cost analysis	Findings (study assumptions)
*Attic insulation*		
Maher (2013)	Cost per kWh saved	Cost per kWh saved: US$0.12 *(equipment lifetime of 10 years; average rebate amount of US$361; and discount rate = 5%)*
Suter and Shammin (2013)*	Internal rate of return	Lifetime internal rate of return: 17.0% *(equipment lifetime of 10 years; gas price per ccf of US$0.78; 151 days in heating seasons; no change in gas price)*
*Central air conditioning*		
Maher (2013)	Cost per kWh saved	Cost per kWh saved: US$0.05 *(equipment lifetime of 10 years; average rebate amount of US$550; discount rate = 5%)*
*Electric heat pumps*		
Alberini et al. (2016)	CO_2_ abatement cost	Cost per ton of CO_2_ eliminated due to rebate: US$46.24 *(equipment lifetime of 10 years; US$450 rebate; 0.608* *kg CO*_*2*_ *emissions avoided per kWh saved)*
*EEM bundle*		
Beagon et al. (2018)	Annual energy savings	Annual energy savings: €72.62 *(€0.06 per kWh)*
Fowlie et al. (2018)*	Lifetime energy savings	Lifetime energy savings: US$2920 per household
		Lifetime internal rate of return: −2.3%
	Private ROI	Cost per ton of CO_2_ eliminated: US$201
	CO_2_ abatement cost	*(equipment lifetime of 16 years, the average life‐span for installed measures; electricity price of US$0.11/kWh and gas price of US$10.46/MMBtu; gas assumed to emit 117 lbs CO*_*2*_ *per MMBtu and electricity assumed to emit 1.87 lbs CO*_*2*_ *per kWh; discount rate = 3%)*
James and Ambrose (2017)	Daily energy savings	Daily gas savings: AUS$0.165 per household
		Daily electricity savings: AUS$0.306 per household
		Daily total energy savings: AUS$0.866 per household
	Daily CO_2_ abatement cost	Change in daily GHG emissions (gas): −0.51 kgCO_2_e
		Change in daily GHG emissions (electricity): −1.33 kgCO_2_e
		Change in daily GHG emissions (total energy): −3.84 kgCO_2_e *(electricity price of AUS$0.29/kWh and gas price of AUS$0.018 cents/MJ; gas assumed to emit greenhouse gas (GHG) emissions of 1.26 kgCO*_*2*_ *‐e/kWh and electricity assumed to emit 0.0039 kgCO*_*2*_ *‐e/MJ)*
Liang et al. (2018)	Cost per kWh saved	Cost per kWh saved: US$0.434 *(US$0.1073 per kWh)*
*Lighting*		
Carranza and Meeks (2016)	First‐year energy savings	First‐year energy savings: US$7.20 per household *(electricity price of US$0.02/kWh; this estimate does not include any aggregate impacts in electricity reliability)*

Alberini et al. (2019) estimate an internal rate of return for installing windows and or insulation, but that is not the primary outcome for this review and the authors do not use the preferred model for this review. To be consistent with other studies, the Fowlie et al. consumption impacts reported in this review are for gas only. However, the only cost analysis conducted by the authors uses total energy (gas + electricity), and so those are the calculations reported in this table. Fowlie et al. also estimate an average rate of social return of −7.8%, which use energy‐avoided marginal costs, rather than energy retail prices and includes the monetised value of the avoided emissions.

* Indicates low risk of bias.

Consistent with the approach taken in this review, we focus on the two studies that report cost analyses and were appraised as low overall risk of bias.

These two studies estimated substantially different rates of return. One of the studies (Fowlie et al., 2018) examined EEM bundles in a large sample of low income homes and found a small negative rate of return and a cost per ton of CO_2_ eliminated that was several times higher than typical estimates of the social cost of CO_2_. The other study (Suter & Shammin, 2013), found a high positive rate of return from installing attic insulation in a small sample of college residential houses. These estimates are consistent with the impact estimates—Fowlie et al. found that installing these relatively expensive bundles led to smaller reductions in energy consumption, while Suter et al. found that installing relatively cheap attic insulation led to substantial reductions in energy consumption.

## 6 DISCUSSION

### 6.1 Summary of main results

#### 6.1.1 Characteristics of included studies

Our database search and grey literature search retrieved 13,629 studies. Independent screening by two reviewers identified 16 eligible studies evaluating 15 different programmes or REEIs. Most studies were conducted in high‐income countries, including five in EU‐28 countries and five in the United States. Three studies were conducted in Australia/New Zealand, and three studies in lower‐middle and low‐income countries (Ethiopia, Ukraine, and Kyrgyz Republic).

The studies evaluated diverse interventions. Half of the studies (*n *= 8) evaluated a bundle of two or more EEMs, that it is, a combination of EEMs that typically varied by household with a single effect size estimating the impact of the whole bundle. The remaining eight studies either evaluated one type of EEM (Carranza & Meeks, 2016; Costolanski et al., 2013; Suter & Shammin, 2013), or reported separate effect sizes for each EEM evaluated (Adan & Fuerst, 2016; Hamilton et al., 2016).

To answer Research Question 3, we collected information on funding mechanisms. In most of the studies REEI installation was subsidised (or partially subsidised), regardless of whether the intervention targeted low‐income households (*n *= 4) or mixed‐income households (*n *= 11); the remaining study (Suter & Shammin, 2016) fully subsidised the renovation of university student housing. Most of the subsidies were provided by governments (*n *= 10) or private organisations (*n *= 4), while the other two studies were funded by the university and researchers who led the evaluation. In 9 of the 15 studies involving households (Suter & Shammin, 2016 involved university students) the subsidies fully covered the REEI cost; in the remaining studies partial subsidies usually covered between 20% and 30% of the total cost. We did not find much information on programme implementation.

Roughly a third of the included studies (*n *= 5) reported that the intervention required an energy audit before the installation of the upgrades. Some of the households in the other studies might have conducted an audit, but this information was not reported.

To answer Research Question 4, we collected data on costs. Nine of the studies provided some information on intervention cost, which for heating/cooling REEIs was $3089 on average with a median of $2348. In Costolanski et al., 2013 the consumer cost was around $0.40 for an 11 W CFL bulb (the market price for the same bulb was $1.42); in Carranza and Meeks (2016) the programme cost was on average $8.81 per household.

All of the included studies reported at least one energy consumption outcome. In most of the studies the data were collected from administrative records or obtained from utility companies and gas and electricity meter operators, including household level energy bills.

#### 6.1.2 Quantitative synthesis

Our quantitative synthesis finds promising evidence for subsidising EEM bundles—the average reduction in energy consumption was statistically significant. The evidence examining individual EEMs is more limited and studies typically find smaller, statistically significant reductions in consumption; a few studies estimate large impacts or negligible impacts. One eligible study (James & Ambrose, 2017) was not included in the synthesis, as the authors did not report enough information to calculate an effect size, and did not respond to a request for this information.

The results were similar when limited to the five low risk of bias studies, with the caveat that the evidence examining any REEI was only one or two studies. This limited evidence and the importance of contextual factors means we could not formally rate the effectiveness of individual REEIs.

To be most helpful for policymakers, funders, agencies, and households who are often choosing between different types of EEMs, we grouped similar interventions together when synthesising findings. Because climate directly determines the amount of energy needed for heating/cooling REEIs, for these REEIs we report effects separately for warmer/colder climate subgroups as well as provide an overall effect across all included studies.

The two included studies evaluating subsidisation of CFLs both found a statistically significant reduction in electricity consumption. The estimated impacts in Carranza and Meeks (2016) had a lower risk of bias and were roughly one‐third of Costolanski et al. (2013), possible due to fewer installed CFLs and less selection bias.

Overall, the four included studies found that installing loft/attic insulation had mixed impacts, with an average impact close to zero. One low risk of bias study conducted in a colder climate—and likely to have installed thicker installation—reported a large reduction in energy consumption (Suter & Shammin, 2013) but had a small sample size (24 households). Another study (Maher, 2013), conducted in a warm, humid climate, reported a statistically significant reduction in consumption. The other two studies, the first of which was low risk of bias, reported smaller effects, but the average amount of additional insulation installed in these studies appears to be minimal.

Two studies examined the replacement or installation of heat pumps (Alberini et al., 2016; Grimes et al., 2016). Replacing older pumps with more efficient versions significantly reduced electricity consumption in a colder climate (Alberini et al., 2016). However, in warmer climates where electric heat pumps were typically installed to replace other heating appliances, Grimes et al. found smaller, positive effects, indicating an increase in energy usage, partially due to households using the heat pumps to cool the residence in warmer weather. However, the Grimes et al. results did not accurately capture heat pumps' impact on total energy consumption, as the authors did not measure coal and wood energy consumption, which are primary energy sources in New Zealand. Supplementary analyses indicate that the installed heat pumps actually reduced overall energy consumption across all sources (i.e., households used more electricity but less unmeasured wood and coal). Thus, the Alberini et al. study impacts are likely to provide more relevant estimates of how heat pumps impact energy consumption.

Three other EEMs were examined by a single study. For instance, Maher (2013) found that in a warm, humid climate, replacing central air conditioning unit with a high‐efficiency unit significantly reduced electricity consumption. A low risk‐of bias study, Adan and Fuerst (2016) conducted independent evaluations of both cavity wall insulation and gas boiler replacement finding significant but relatively smaller reductions in gas.

Eight studies examining EEM bundles—combinations of two or more EEMs, with the specific EEMs installed differing by household—typically found promising results. There was significant variation in impacts, possibly due to variation in risk of bias, population and the EEMs installed. Reductions in energy consumption were statistically significant in seven of the eight studies. Focusing on the two low risk of bias studies, conducted with mostly low‐income households, the impact on residences with installed bundles was statistically significant.

One low risk of bias study (Adan & Fuerst, 2016) examined the impact of installing identical bundles in each dwelling and found that the impact of installing additional EEMs does not appear to be additive. The authors attribute the nonadditivity to the prebound effect—households in the least efficient residences used behaviour to consume less energy (prebound effect) and were most likely to install multiple EEMs, and their low‐consumption behaviour continued and minimised impacts.

### 6.2 Overall completeness and applicability of evidence

Investors and other funders are spending billions of euros and dollars to install REEIs, hoping to reduce energy consumption, energy poverty, and GHG emissions. These expenditures are often justified based on predicted savings, but several studies find that actual savings from installing REEIs are typically lower than predicted (Fowlie et al., 2018; Gillingham et al., 2013; Grimes et al., 2016; Howden‐Chapman et al., 2007). Given the resources devoted to REEIs and their importance in mitigating climate change, solid evidence of their impacts and additional rigorous impact evaluations (experimental and quasi‐experimental studies) are needed. For example, several important REEIs, such as passive cooling systems, heating controls, and information provision were examined by only one study or not at all.

Future studies should also broaden the contexts in which REEIs are examined. Regulations, climate, construction methods vary by country, and accordingly the impact of REEIs will likely vary by region. We did not identify any studies in South America or and only one study each in Africa and Asia, both involving CFLs. The latter two regions are projected to experience significant population growth and increases in housing and energy demand, increasing the need for evidence. Moreover, households in these regions will increasingly require cooling interventions, and we did not find any study on passive cooling systems or district cooling (and only one study on central air conditioning). We also did not identify any studies in cold climates, such as Finland or Canada, and the only study occurring in a moderate climate (James & Ambrose, 2017) did not provide data that enabled the calculation of impacts.

Finally, we recognise that numerous studies were excluded from this review for not using an eligible methodology but still provide rich qualitative and quantitative insights about REEI effects. For example, these studies explain how context, population, and implementation shape REEI impacts.

### 6.3 Quality of the evidence

Most of the included studies, especially the quasi‐experiments (9 of 11), were appraised as high overall risk of bias. These studies typically used difference‐in‐differences methods, especially fixed‐effects regression (eight studies), to control for time‐invariant differences between households. These are rigorous designs, but authors often did not match comparison participants or use other methods to control for selection bias. Household decisions to install REEIs are plausibly time‐dependent and vary in ways that could also impact outcomes. For example, households that become more environmentally conscious or add members might simultaneously decide to install REEIs and change their energy consumption.

In general, the randomised trials were well‐implemented and three of five were appraised as low risk of bias. One of the two other studies (Carranza & Meeks, 2016) might have been appraised as low risk of bias, but the authors did not describe some sample attrition and did not respond to a request for information; the study was otherwise well‐implemented. The other high risk of bias randomised trial (James & Ambrose, 2017) compromised random assignment by assigning some households partially based on perceptions of responsiveness.

Low risk of bias studies provide the most reliable evidence of how REEIs affect energy consumption, but high risk of bias studies can be more appropriate when there are limitations with the low risk of bias studies examining an REEI. For example, Grimes et al. (2016) did not measure changes in the primary heating sources used in New Zealand (such as wood and coal), changes which could have impacted overall energy efficiency. Thus, although Alberini et al. (2016) had a higher risk of bias rating, the study might actually provide more relevant estimates for how more efficient heat pumps affects total energy consumption across all fuels.

### 6.4 Potential biases in the review process

The databases we searched mostly contain studies in English, or studies with abstracts and indexes in English. Our search strategy identified 79 papers using a non‐Latin script, and 46 published in a Latin script but not in English. Screeners were able to either understand the language or translate it to make an informed decision. None of these studies were eligible to be included in the review. Researchers often publish the abstract of their recent papers in English to make sure their studies are read and cited as much as possible, and we assume the risk of missing papers in other languages is low (Boutron et al., 2021).

To minimise bias, every study was independently double screened, and all the included studies had data extracted and risk of bias appraised by two independent researchers, with reconciliation performed by a third core team staff.

### 6.5 Agreements and disagreements with other studies or reviews

We are not aware of other rigorous effectiveness SRs that synthesise the evidence on REEIs and report an effect size. Russell‐Bennett et al. (2019) conducted a SR of studies on household energy efficiency interventions, a broad category including, but not limited to, the installation of REEIs. Like this review, they found that overall energy efficiency interventions reduced electricity consumption, however, this was not systematically calculated. In particular, they found that a multi‐layered approach, including, for instance, the installation of EEMs, combined information and behavioural interventions, has positive effect compared to single interventions. The authors also encountered challenges comparing the impacts from different studies because findings were often not fully described–something we also highlight in the findings section and the research implications section below.

We are aware of other reviews conducted in this field, however, most of them are not comparable to this study work due to differences in interventions, outcomes, and/or methodologies. For instance, Lomas et al. (2018) found that that the effects of heating control systems (an intervention not eligible for this review) depend on consumer behaviour. Munton et al. (2014) found that smart thermostats were not more effective than traditional thermostats on reducing energy consumption, due to inappropriate use of technology. Finally, Maidment et al. (2014) found that improved winter warmth and lower humidity due to EEMs had positive results cardiovascular and respiratory health, and mental health.

## 7 AUTHORS' CONCLUSIONS

Our search identified 16 rigorous impact evaluations and 11 of these studies were rated as having a high risk of bias. We conclude that installing REEIs usually reduces household energy consumption, and note substantial variation in impacts. This variation is likely due to contextual factors, such as: the populations involved, how the EEMs are installed, the specific EEMs installed, and how the EEMs affect household behaviour. Additional high quality impact evaluations that provide more detailed descriptions of installed EEMs are needed to draw stronger conclusions and better understand variation in impacts.

### 7.1 Implications for practice

The overall evidence base included in this SR, including the sub‐set of low risk of bias studies, provide positive evidence that installing REEIs reduces energy consumption. This supports REEIs being an important pillar of policies that aim to reduce residential CO_2_ emissions (such as the European Union's Green Deal^2^ and Renovation Wave^3^).

Implementation and context matters, as the SR found some situations when REEIs might not reduce energy consumption. For example, this was the case when REEI implementation did not follow recommended practice and involved minimal insulation (such as in Hamilton et al., 2016). Similarly, REEIs that provide additional heating and cooling functionality might increase electricity consumption, the “rebound” effect (such as in Grimes et al., 2016). In addition, Alencastro et al. (2018) highlight the importance of preventing quality defects when installing EEMs, as such defects might lead to different building energy performance.

Aside from the CFLs, the high costs of installing the EEMs (between US$900 and US$6000) would probably deter many households, especially low‐income households, from installing EEMs without subsidies. Subsidies can be justified economically (Cattaneo, 2019) as some of the benefits, such as pollution reduction, do not directly accrue to the households (i.e., there are positive externalities).

Information on costs varied among the eight studies that reported a cost analysis, depending to a large extent on how costs were calculated and on the type of intervention. Some studies estimated that REEI interventions led to cost savings, but others identified small or negative cost‐effectiveness.

The SR only identified one study (Howden‐Chapman et al., 2007, low risk of bias) that examined health outcomes as well as energy consumption. This study found that REEIs had consistent positive impacts on self‐reported physical and mental health outcomes (for instance self‐reported vitality, happiness, winter colds or flu). Other studies, not eligible for this SR because they did not report energy consumption outcomes, also examine health outcomes (i.e., Allcott & Kessler, 2019; Francisco et al., 2017; Howden‐Chapman et al., 2007; Osman et al., 2010; and many other studies available through the Energy Efficiency EGM^4^).

Despite this positive evidence, several studies indicate that installing REEIs often reduces energy consumption less than prediction models estimate (Fowlie et al., 2018; Grimes et al., 2016; Howden‐Chapman et al., 2007). Accordingly, practitioners should incorporate evidence from low risk of bias studies when predicting impacts of REEIs.

### 7.2 Implications for research

Given the limited high quality evidence evaluating REEIs, more well‐implemented randomised trials and rigorous quasi‐experiments are needed. These studies should be conducted in more countries; currently no studies examine the impact of altering insulation or heating/cooling systems in Africa, Asia or South America. More research is also needed on other building EEMs, as a recent evidence gap map found that few studies examine government, public or commercial buildings (Berretta et al., 2021). There is debate about the barriers to randomised evaluations in the energy efficiency space (Cooper, 2018; Vine et al., 2014). Even when randomised evaluations are not feasible, natural experiments and quasi‐experimental methods can provide useful causal evidence (Cooper, 2018).

This SR identifies variation in REEI impacts. Some of this variation is likely due to unreliable study methods, as only 5 of the 16 included studies were assessed as having a low risk of bias. Previous work has concluded that counterfactual designs are “much rarer in environmental policy than in other social policy fields” (Ferraro, 2009, p. 78). Future work could use reporting guidelines or evidence standards from other domains (such as Cochrane's RoB 2 or the Clearinghouse for Labor Evaluation and Research) to identify ways to reduce risk of bias. However, there was important variation even within low risk of bias studies.

To inform future programmes, research should systematically seek to understand the causes of that variation. Studies should examine how factors, such as preinstallation energy audits or government regulations, influence REEIs' impact. Research can rigorously study these factors (moderators) by experimentally manipulating the REEI features that households receive or quasi‐experimentally using observational data. Additionally, once there is a larger literature evaluating diverse REEIs, syntheses can use meta‐regression to examine how impact variation is associated with different factors.

Future work should also describe the baseline residences and interventions in more detail—existing studies often do not provide enough information to understand the EEMs implemented. For example, future studies could report the type and amount of insulation installed and the efficiency ratings of original and replacement boilers. When there is variation, such as different households receiving different amounts of insulation, the variance, range, and frequencies can be provided. Finally, studies examining REEI bundles should report the percentage of households installing each bundle and estimate impacts by bundle subgroup if there is sufficient statistical power.

## ADVISORY GROUP MEMBERS

Gesche Huebner: University College London.

James Milner: London School of Hygiene and Tropical Medicine.

Nicola Willand: Royal Melbourne Institute of Technology & Melbourne Technical College.

Marina Economidou‐European Commission, Joint Research Centre.

Claire Walsh: J‐PAL, King Climate Action Initiative.

Jan Minx: Mercator Research Centre on Global Commons and Climate Change.

## CONTRIBUTIONS OF AUTHORS

Content: Miriam Berretta, Joshua Furgeson, Ian Hamilton, and Yue (Nicole) Wu.

Systematic review methods: Joshua Furgeson, Collins Zamawe, and Miriam Berretta.

Statistical analysis: Joshua Furgeson.

Information retrieval: John Eyers.

## DECLARATIONS OF INTEREST

There are no potential conflicts of interest.

## SOURCES OF SUPPORT

External Sources

European Investment Bank.

## PLANS FOR UPDATING THIS REVIEW

There are no plans to update this review at the moment.

## DIFFERENCES BETWEEN PROTOCOL AND REVIEW

We were not able to study the following outcomes because no study reported them: energy security, air quality index, income savings, GHG emissions, job creation, building stock value.

We did not conduct the subgroups analyses on resident socioeconomic status or on the source of the funds used for the intervention because few studies provided this information.

We did not conduct a funnel analysis due to the small number of effects (no analysis included more than seven effects).

We changed the Research Question 3 from “For the included studies, what are the programme design, implementation, context, and funding mechanisms?” to “For the included studies, what are the implementation, context, and funding mechanisms?” because we were not able to collect much information on programmes design. We did not find much information on implementation and context.

## Supporting information

Supporting information.

## Footnotes

1 We retrieved the HHD from www.degreedays.net

2 European Green New Deal: https://ec.europa.eu/info/strategy/priorities-2019-2024/european-green-deal_en

3 Renovation Wave: https://ec.europa.eu/energy/topics/energy-efficiency/energy-efficient-buildings/renovation-wave_en

4
https://gapmaps.3ieimpact.org/evidence-maps/energy-efficiency-egm
