# Supplementary material for: Residential energy efficiency interventions: A meta‐analysis of effectiveness studies
Source: Campbell Syst Rev. 2021 Dec 17;17(4):e1206. doi: 10.1002/cl2.1206 (PMC8988770; doi:10.1002/cl2.1206)
Supplement: Supplementary file 1 — Supporting information. [file CL2-17-e1206-s001.docx]

# Appendices

# Appendix A. Data Extraction Template

***A1. Descriptive / qualitative coding tool***

| Description | Question | Coding |
| --- | --- | --- |
| Unique study ID | What is the EPPI ID for the record? | For example, 44126990 |
| Reviewer name | Who completed the data extraction? | Open answer |
| Document type | Is this the main paper of a linked document? | Report 'main paper' or 'linked document' |
| Main paper study ID | If linked document, provide EPPI ID for the main paper | ID number |
| Time taken to complete extraction | How many minutes did it take to code the study?   We are genuinely interested in understanding how long it takes. We completely understand that some studies take longer to code. There are no repercussions for reporting any length of time so try to report truthfully - this helps us with planning future work, and means we budget correctly to involve consultant teams. | Mins |
| Form of publication | What is the impact evaluation publication type? | 1 = Peer-reviewed journal |
|  |  | 2 = Book chapter/book |
|  |  | 3 = Conference paper |
|  |  | 4 = Organisation report 5 = Working-paper |
|  |  | 6 = Implementation document |
|  |  | 7 = Website |
|  |  | 8 = Other grey (specify) |
|  |  | 9 = PhD thesis / dissertation |
| Publication year | What is the publication year? | XXXX |
| Study funding / Funding agency | Who is funding the evaluation/study? | 1 = Public institution (e.g. gov, university, research institute) |
|  |  | 2 = Private institution (e.g. private firm) |
|  |  | 3 = Multilateral Organisation (e.g. World Bank, UN) |
|  |  | 4 = Foundations |
|  |  | 5 = NGO |
|  |  | 999 = Not clear |
|  |  | 9 = Not applicable (Non-funded) |
| Name of study funding agency | Please add name of the agency funding the evaluation | Open answer |
| Study setting (World Bank) | Select the World Bank income classification of the country at the time of the study | 1 = Low income country |
|  |  | 2 = Lower-middle income country |
|  |  | 3 = Upper-middle income country |
|  |  | 4 = High income country |
| Region (World Bank) | Select region(s) the study was conducted in according to World Bank. For more info on region classification see http://data.worldbank.org/country | 1 = East Asia & Pacific |
|  |  | 2 = Europe & Central Asia |
|  |  | 3 = Latin America & Caribbean |
|  |  | 4 = Middle East & North Africa |
|  |  | 5 = South Asia |
|  |  | 6 = North America |
|  |  | 7 = Sub-Saharan Africa |
| Country | List country/countries the study was conducted in | Country 1, Country 2, etc. |
| Detailed location | If provided, give detailed information on where the study took place within a country, for example regions/districts covered | Open answer |
| Independence of evaluation | What level of independence is there between the implementing agency and study team? | 1 = Funding and author team independent of implementers/ funders of programme.  i.e. The organisation that funded the evaluation, and the evaluation authors, are independent from those responsible for implementing the programme. |
|  |  | 2 = Funding independent of implementers/ funders of programme, but includes authors from funder/ implementer  i.e. The evaluation was not funded by those responsible for implementing the programme. But the evaluation includes authors from either the funding agency of the implementation team. |
|  |  | 3 = Evaluation funded and undertaken by funders/ implementers.  i.e. The evaluation was funded and undertaken by those responsible for implementing the programme. |
|  |  | 8 = Unclear |
| Independent data collection | Has the data been collected by an independent organisation (a different one from the implementer organisation)? | 1 = Yes |
|  |  | 2 = No |
|  |  | 8 = Not clear |
| Conflict of interest | Is there a potential conflict of interest associated with study which could influence results collected/reported? (e.g. Is there a declaration of conflict of interest?  Is any of the authors related in any way to the funding or implementing institution?) | 1 = Yes |
|  |  | 2 = No |
| Comments on conflict of interest | Please add reason for your answer to whether there is a conflict of interest. | Open answer |
| Language of publication | Language of publication of the impact evaluation, e.g. Spanish, English etc. | Open answer |
| Other methods | If the impact evaluation addresses other questions than effectiveness - note questions and methods used here. | Open answer (this will include for example mixed-methods to assess implementation, adherence, participant views etc.) |
| Baseline socio-economic status of participants | Report any data / description on baseline socio-economic status of participants. | Open answer |
| Unit of observation | What is the unit of observation for the analysis? | Open answer, include whether group are residential, commercial or community. |
| Total number of units | State the total number of units, if possible by treatment and control groups. | Open answer |
| Age groups within sample | What is the age group of respondents within the sample?   When the unit of analysis is not an individual, report the most common group, e.g. a school would focus on children. | 1 = Children <18 years |
|  |  | 2 = Adults ≥18 |
|  |  | 3 = Both children and adults |
|  |  | 8 = Not clear |
|  |  | 9 = Not applicable |
| Sex | What is the sex of the sample? | 1 = Males only |
|  |  | 2 = Females only |
|  |  | 3 = Mixed |
|  |  | 8 = Not clear |
|  |  | 9 = Not applicable |
| Income level | Are the beneficiaries low income households? | 1= yes 2 = no 999 = Unclear |
| Other useful information | Report any other characteristics relating to the study sample. | Open answer |
| Control/comparison scenario | What intervention did the control/comparison group receive? | 1 = No treatment |
|  |  | 2 = Business as usual (specify) |
|  |  | 3 = Alternative intervention (specify) |
|  |  | 4 = Other (specify) |
|  |  | 999 = Not clear |
| Intervention name | State the programme or project name. If no name, then list the location | Open answer |
| Intervention description | Provide descriptive details about the intervention in your own words.  Aim to provide up to 2-3 sentences, although expect to provide more or less content depending on the complexity of the intervention. | Open answer |
| Specific objectives of intervention | State any objectives stated in study or other document.  Please directly copy and paste the objectives stated in the document. | Open answer |
| Intervention activities | Is the intervention comprised of one or more activities? | 1 = Single service / activity |
|  |  | 2 = Multiple services / activities |
|  |  | 999 = Unclear |
| Intervention type | What are the activities the intervention is comprised of? | 1 = Wall/roof/floor cavity insulation |
|  |  | 2 = Loft/attic insulation |
|  |  | 3 = External/internal wall insulation |
|  |  | 4 = Replacement (oil or gas) boiler or furnace |
|  |  | 5= Heating controls |
|  |  | 6 = Passive cooling system and design |
|  |  | 7 = EE lighting (i.e. CFL, LED) |
|  |  | 8 = Window and door upgrades |
|  |  | 9 = District heating/cooling systems |
|  |  | 10 = Energy audits + EE improvements |
|  |  | 11 = Behaviour change interventions+ EE improvements |
|  |  | 999 = Unclear |
|  |  |  |
| Intervention funding agency | Type of funder | 1=Government |
|  |  | 2=NGO |
|  |  | 3=Multilateral/bilateral organisation |
|  |  | 4= Foundation |
|  |  | 5= Private sector |
|  |  | 6= Other |
| Intervention funding agency | Name of intervention funding agency | Open answer |
| Setting of the intervention | Where is the intervention delivered? | 1 = Rural |
|  |  | 2 = Urban |
|  |  | 3 = Both |
|  |  | 999 = Unclear |
| Intervention scale | At what level is the intervention being delivered at? | 1 = National delivery |
|  |  | 2 = Regional (delivery in one or more regions) |
|  |  | 3 = Local level (delivery in one or more sub-regions) |
|  |  | 999 = Unclear |
| Other useful information | Report any other characteristics relating to the intervention. | Open answer |
| Targeting methods | How were beneficiaries selected for the programme (E.g.: how was the targeting implemented)? | Open answer |
| Intervention start | Start date of intervention, if no stated write 999 | XX/XXXX |
| Intervention end | State end date (if ongoing state ongoing) | XX/XXXX |
| Intervention length | Start intervention length (months) | State number of months |
| Consideration of equity | Does the study consider equity? | 1 = Yes 2 = No |
| Equity methods | Does the study explicitly consider equity in any of the following ways? | 1=  Intervention targets vulnerable population |
|  |  | 2 =  Sub-group analysis by sex |
|  |  | 3 =  Sub-group analysis (other than sex) |
|  |  | 4 =  Heterogeneity analysis (other than sub-group) |
|  |  | 5 =  Equity sensitive analytical framework |
|  |  | 6 =  Equity sensitive methodology |
|  |  | 7 =  Equity sensitive research process |
|  |  | 8 =  Measures effects on an inequality outcome |
|  |  | 9 = Research ethics informed by equity |
|  |  | 10 = Not applicable |
| Equity dimension | If answered 1-3 in "Equity methods" What dimension(s) of equity does the study consider? Report "N/A" otherwise.  PROGRESS + indicators (multiple choice - may pick more than one). For more information, please see:  https://www.jclinepi.com/article/S0895-4356(13)00334-X/pdf. | 1 = place of residence |
|  |  | 2 = race / ethnicity |
|  |  | 3 = occupation |
|  |  | 4 = sex |
|  |  | 5 = religion |
|  |  | 6 =education |
|  |  | 7 = socioeconomic status |
|  |  | 8 = social capital |
|  |  | 9 = age |
|  |  | 10 = disability |
|  |  | 11 = sexual orientation |
| Information about programme take-up | Is there any information about programme take-up?  Commentary by authors should be used when information on programme take / up etc. is not backed up by some sort of research / when the authors do not report that/how they collected data to assess these areas. | Open answer. |
|  |  |  |
|  |  |  |
| Methods of assessing take-up | Which methods are used to assess programme take-up? | 1= Observation by intervention staff |
|  |  | 2= Reporting by participants |
|  |  | 3= Other |
|  |  | 4= Commentary from author |
|  |  | 999= Not measured, N/A |
| Results of the assessment of take-up | What is the result/ information provided of the assessment of programme take-up? If the programme implementation did not permit beneficiaries to accept the intervention then report this. | Open answer. |
| Outcome type | What outcomes were assessed? | 1 = Primary outcomes |
|  |  | 2 = Secondary outcomes |
| Net energy savings or consumption changes | Were any of the following outcomes assessed (multi code okay)? | 1 = rates of energy saved |
|  |  | 2 = changes in consumption used |
|  |  | 3 = Other (specify) |
| Energy security | Were any of the following outcomes assessed (multi code okay)? | 4 = beneficiaries can now pay the energy bill |
|  |  | 5 = beneficiaries can afford to keep higher temperature in the houe/apartment in winter |
|  |  | 6 = Other (specify) |
| GHG emissions | Were any of the following outcomes assessed (multi code okay)? | 7= changes in GHG emissions rates |
| Air quality indices and pollution (rates) | Were any of the following outcomes assessed (multi code okay)? | 8 = changes in air quality rates in the house/apartment |
|  |  | 9 = changes in air quality rates in the area (e.g. city, region) |
|  |  | 10 = Other (specify) |
| Income savings | Were any of the following outcomes assessed (multi code okay)? | 1 = cheaper energy bills |
|  |  | 2 = Other (specify) |
| Health status, comfort, and wellbeing | Were any of the following outcomes assessed (multi code okay)? | 3 = Mortality rate |
|  |  | 4 = Sickness rate |
|  |  | 5 = Mental health rates |
|  |  | 6 = Other (specify) |
| Job creation | Were any of the following outcomes assessed (multi code okay)? | 7 = No. of jobs created |
|  |  | 8 = Other (specify) |
| Building stock value | Were any of the following outcomes assessed (multi code okay)? | 9 = Reported increases of the building/house value |
|  |  | 10 = Other (specify) |

***A2. Quantitative data coding tool***

|  | Description | Question | Coding |
| --- | --- | --- | --- |
| ID | Unique study identification # |  | For example, SC001 |
|  | First author - impact evaluation | Surname | Open answer |
| Outcome for effect size (answer for all studies) | Outcome | Which outcome is being coded? | 1 = Knowledge and attitudes about services 2 = Access to services 3 = Use of services 4 = Performance or quality of services 5 = Income or poverty status 6 = Health outcome 7 = Nutritional status / food security 8 = Resilience (including coping strategies) 9 = Environmental (non-human) outcome 10 = Social and psychological outcomes 11 = Other |
|  | Definition of outcome | Please provide the authors definition of the outcome (including description of the sub-group if relevant) | Open answer |
|  | Follow up period | What is the follow up period of this outcome after initiation of intervention? (specify in months) | Number of months |
|  | Sub-group analysis | Is this effect size data for a sub-group? | 1 = No 2 = Yes |
|  | Sub-group analysis description | If yes to question 2, which type of sub-group? | Open answer - this can include separate samples for gender, income, place of residence |
|  | Effect size location | Which page(s) contain the effect size data? | Open answer |
|  | Data to be extracted | Which type of data to be extracted? | 1 = Continuous - means and SDs 2 = Continuous - mean difference and SD 2 = Dichotomous outcome - proportions 3 = Regression data |
| Effect size data (answer for all studies) | Sample size metric | Sample size unit of analysis | 1= Individual 2= Household  3= Group (e.g. community organisation) 4= Village 5 = Other 6 = Not clear |
|  | Treatment effect estimated | What treatment effect is estimated? | 1=ITT  2=ATET  3=ATE  4=LATE |
|  | Sample size (treatment) | Initial sample size treatment group | # |
|  | Sample size (control) | Initial sample size control group | # |
|  | Sample size (total) | Initial sample size total | # |
|  | Observations (treatment) | Number of treatment observations after attrition / follow up | # |
|  | Observations (control) | Number of control observations after attrition / follow up | # |
|  | Observations (total) | Total number of control observations after attrition / follow up | # |
| Outcome data - if continuous (Means and SDs) | Baseline outcome treatment | State result of baseline outcome for treatment group | # |
|  | SD Baseline outcome treatment | State SD of baseline outcome measure for treatment group | # |
|  | Baseline outcome control | State result of baseline outcome for control group | # |
|  | SD Baseline outcome control | State SD of baseline outcome measure for control group | # |
|  | Outcome in treatment post intervention | State result of post intervention outcome for treatment group | # |
|  | SD Outcome in treatment post intervention | State SD of post intervention outcome measure for treatment group | # |
|  | Outcome in control post intervention | State result of post intervention outcome for control group | # |
|  | SD Outcome in control post intervention | State SD of post intervention outcome measure for control group | # |
|  | Outcome in treatment 1st follow up | State result of 1st follow up outcome measure for treatment group | # |
|  | SD Outcome in treatment 1st follow up | State SD 1st follow up outcome measure for treatment group | # |
|  | Outcome in control 1st follow up | State result of 1st follow up outcome measure for treatment group | # |
|  | SD Outcome in control 1st follow up | State SD 1st follow up outcome measure for treatment group | # |
| Outcome data - If continuous (Mean difference and SD / SE at follow up) | Mean difference at follow up | State mean difference | # |
|  | SD at follow up | State SD at follow up | # |
|  | SE | State SE | # |
| Outcomes data - if dichotomous (Proportions r) | Baseline number with outcome in treatment | State result of baseline outcome for treatment group | # |
|  | Proportion with outcome at baseline in treatment | State proportion with outcome at baseline in treatment | # |
|  | Baseline number with outcome in control | State result of baseline outcome for treatment group | # |
|  | Proportion with outcome at baseline in control | State proportion with outcome at baseline in control | # |
|  | Number with outcome in treatment post intervention | State number with outcome post intervention for treatment group | # |
|  | Proportion with outcome in treatment group post intervention | State proportion with outcome post intervention in control group | # |
|  | Number with outcome in control post intervention | State number with outcome post intervention for control group | # |
|  | Proportion with outcome in control group post intervention | State proportion with outcome post intervention in control group | # |
|  | Number with outcome in treatment 1st follow up | State number with outcome at 1st follow up for treatment group | # |
|  | Proportion with outcome in treatment group 1st follow up | State proportion with outcome at 1st follow up in treatment group | # |
|  | Number with outcome in control 1st follow up | State number with outcome at 1st follow up for control group | # |
|  | Proportion with outcome in control group 1st follow up | State proportion with outcome at 1st follow up in control group | # |
| Regression data | OLS | OLS used? | 1=Yes 2=No |
|  | Logistic | Logistic used? | 1=Yes 2=No |
|  | Type of logistic | What type of logistic regression? | 1=binomial 2=multinomial |
|  | GLS/WLS | GLS or WLS used? | 1=Yes 2=No |
|  | other regression types | Other regression type used? Specify | open answer |
|  | continuous outcome | Is the outcome continuous? | 1=Yes 2=No |
|  | dichotomous outcome | Is the outcome dichotomous? | 1=Yes 2=No |
|  | multiple outcome categories | Does the outcome have more than 2 categories? | 1=Yes 2=No 3=Continuous |
|  | type of coefficient | What is the coefficient type? | 1=raw 2=standardized 3=other |
|  | coefficient | What is the coefficient estimate? | # |
|  | pooled standard deviation of outcome | What is the pooled standard deviation of the outcome? | # |
|  | standard error | What is the standard error of the coefficient estimate? | # |
|  | t test | What is the t statistic associated with the focal predictor? | # |

# Appendix B: Risk of bias appraisal tool

This tool classifies study design and assesses the risk of bias for quantitative impact evaluations. This appendix provides an overview of the critical appraisal domains and criteria. Additional detail is available from the corresponding author.

| Description | Question | Coding |
| --- | --- | --- |
| Unique study identification # | Study | For example, SC001 |
| Paper | Surname / year of first author of paper for effect size data extraction | Open answer |
| Design type | What type of study design is used? | 1= Randomised controlled trial (RCT) (random assignment to households/individuals) or quasi-RCT 2= Cluster-RCT (quasi-RCT) 3= Natural experiment: randomised or as-if randomised  4= Natural experiment: regression discontinuity (RD) 5 = CBA (non-randomised assignment with treatment and contemporaneous comparison group, baseline and endline data collection) – individual repeated measurement  6= CBA pseudo panel (repeated measurement for groups but different individuals) 7= Interrupted time series (with or without contemporaneous control group)  8= Panel data, but no baseline (pre-test) 9 = Comparison group with endline data only |
| Methods used for analysis | Which methods are used to control for selection bias and confounding? | 1= Statistical matching (PSM, CEM, covariate matching)  2= Difference in differences (DID) estimation methods 3= IV-regression (2-stage least squares or bivariate probit) 4=Heckman selection model 5= Fixed effects regression 6= Covariate adjusted estimation  7= Propensity weighted regression 8= Comparison of means 9 = Other |
| Design and analysis method description | Briefly describe the study design and analysis method undertaken by the authors | Open answer |
| Unit of analysis | Is unit of analysis in cluster allocation addressed in standard error calculation (RCT and NRS)? | 1=Yes 2=No 3=Not reported/unclear 4=Not applicable |
| Method used to address differences between UoA and unit of data collection | Briefly describe methods used to adjust standard errors to account for correlation of observations within clusters (e.g. cluster-robust standard errors reported) | Open answer |
| Type of comparison group | Indicate type of comparison group | 1=No intervention (service delivery as usual) 2=Other PITA intervention 3=Pipeline (wait-list) control (still service delivery as usual) |
| Assignment mechanism | 1: Mechanism of assignment: was the allocation or identification mechanism random or as good as random? | 1= Yes, 2 = Probably Yes, 3 = Probably No, 4 = No, 8 = Unclear |
| Assignment justification | Justification for coding decision  (Include a brief summary of justification for rating, mentioning your response to all sub questions, cite relevant pages) | Open answer |
| Confounding | Group equivalence: was the method of analysis executed adequately to ensure comparability of groups throughout the study and prevent confounding | 1= Yes, 2 = Probably Yes, 3 = Probably No, 4 = No, 8 = Unclear |
| Confounding justification | Justification for coding decision  (Include a brief summary of justification for rating, mentioning your response to all sub questions, cite relevant pages) | Open answer |
| Selection bias | Was any differential selection into or out of the study (attrition bias) adequately resolved? | 1= Yes, 2 = Probably Yes, 3 = Probably No, 4 = No, 8 = Unclear |
| Selection bias justification | Justification for coding decision  (Include a brief summary of justification for rating, mentioning your response to all sub questions, cite relevant pages) | Open answer |
| Spill-overs, cross-overs and contamination | 2: Spill-overs, cross-overs and contamination: was the study adequately protected against spill-overs, cross-overs and contamination? | 1= Yes, 2 = Probably Yes, 3 = Probably No, 4 = No, 8 = Unclear |
| Spill-overs justification | Justification for coding decision  (Include a brief summary of justification for rating, mentioning your response to all sub questions, cite relevant pages) | Open answer |
| Motivation bias | Was the process of being observed free from motivation bias (e.g. Hawthorne effects)? | 1= Yes, 2 = Probably Yes, 3 = Probably No, 4 = No, 8 = Unclear |
| Motivation justification | Justification for coding decision  (Include a brief summary of justification for rating, mentioning your response to all sub questions, cite relevant pages) | Open answer |
| Outcome reporting | 3: Outcome reporting: was the study free from selective outcome reporting? | 1= Yes, 2 = Probably Yes, 3 = Probably No, 4 = No, 8 = Unclear |
| Outcome reporting | Justification for coding decision  (Include a brief summary of justification for rating, mentioning your response to all sub questions, cite relevant pages) | Open answer |
| Analysis reporting | 4: Analysis reporting: was the study free from selective analysis reporting? | 1= Yes, 2 = Probably Yes, 3 = Probably No, 4 = No, 8 = Unclear |
| Analysis reporting | Justification for coding decision  (Include a brief summary of justification for rating, mentioning your response to all sub questions, cite relevant pages) | Open answer |
| Performance bias | 5: Performance bias: was the process of being observed free from motivation bias? | 1= Yes, 2 = Probably Yes, 3 = Probably No, 4 = No, 8 = Unclear |
| Performance bias | Justification for coding decision  (Include a brief summary of justification for rating, mentioning your response to all sub questions, cite relevant pages) | Open answer |
| Other bias | 6: Other risks of bias: Is the study free from other sources of bias? Including around measurement of the intervention | 1= Yes, 2 = Probably Yes, 3 = Probably No, 4 = No, 8 = Unclear |
| Other bias | Justification for coding decision  (Include a brief summary of justification for rating, mentioning your response to all sub questions, cite relevant pages) | Open answer |
| Blinded participants | Blinding of participants? | 1=Yes 2=No 9= N/A |
| Blinded observers | Blinding of outcome assessors? | 1=Yes 2=No 9= N/A |
| Blinded analysts | Blinding of data analysts? | 1=Yes 2=No 9= N/A |
| Method used to blind | Describe method(s) used to blind | Open answer (including describe method of placebo control) |

# Appendix C: Search strategy

All the websites were searched in November 2020.

List of additional resources consulted

| **Specific organisations names** | **Link** | **Key words used** | **N. hits retrieved (if applicable)** | **N. hits added in EPPI** | **Comments** |
| --- | --- | --- | --- | --- | --- |
| Collaboration for Environmental Evidence | <https://www.environmentalevidence.org/> | energy efficiency; energy | 2 | 0 | All systematic reviews and only two energy related |
| E2e, group of economists focused on EE and IEs | <http://e2e.haas.berkeley.edu/> | energy efficiency, residential | 46 | 2 |  |
| eceee Summer Study | https://www.eceee.org/library/conference_proceedings/eceee_Summer_Studies/ | energy efficiency | 3 | 0 | Only one relevant result but is a cancelled event |
| Energy consumers Australia | <https://energyconsumersaustralia.com.au/publications/our-research> | energy efficiency | 10 | 0 |  |
| Environmental and Energy Study Institute EESI | <https://www.eesi.org/publications> | energy efficiency | 70 | 0 | White papers category |
| eScholarship University of California | <https://escholarship.org/> | energy efficiency, residential energy efficiency | 69857 | 0 | A lot to go through, in general not so relevant |
| GEF (Global Environmental Facility) evaluation database | <http://www.gefieo.org/evaluations/all> | energy efficiency | 144 | 0 |  |
| Institute for European Energy and climate policy | <http://www.ieecp.org/publications/reports-briefs-and-factsheets/> | energy efficiency | 24 | 0 | Focus on financing |
| Institute of the Environmental and sustainability | <https://www.ioes.ucla.edu/publications/> | energy efficiency; energy | 47 | 0 |  |
| International Energy Agency (IEA) | <https://www.iea.org/> |  | No exact number available | 1 |  |
| International Energy Program Evaluation Conference | <https://www.iepec.org/> | energy efficiency | No exact number available | 10 |  |
| **Evaluation repositories names** | **Link** |  |  |  |  |
| 3ie Repository of IEs | <https://www.3ieimpact.org/> | energy efficiency; residential energy efficiency; energy efficiency intervention; energy saving; energy consumption, energy efficiency measures; | 57 | 0 |  |
| 3ie RIDIE (Registry for International Development IEs): | <https://ridie.3ieimpact.org/> | energy efficiency, residential energy efficiency,energy efficiency intervention, energy saving, energy consumption, energy efficiency measures; | No exact number available | 0 |  |
| African Development Bank (AfDB) | <https://www.afdb.org/en> | energy efficiency, residential energy efficiency,energy efficiency intervention, energy saving, energy consumption, energy efficiency measures | No exact number available | 1 |  |
| Asian Development Bank (ADB) | <https://www.adb.org/countries/myanmar/main> | energy efficiency, residential energy efficiency,energy efficiency intervention, energy saving, energy consumption, energy efficiency measures | No exact number available | 0 |  |
| CARE International | <https://www.care-international.org/> | energy efficiency, residential energy efficiency,energy efficiency intervention, energy saving, energy consumption, energy efficiency measures | 24 | 0 |  |
| Centre for Effective Global Action (CEGA) | <https://cega.berkeley.edu/> | energy efficiency, residential energy efficiency,energy efficiency intervention, energy saving, energy consumption, energy efficiency measures; | No exact number available | 0 |  |
| Centre for Public Impact | <https://www.centreforpublicimpact.org/> | energy efficiency; | 0 | 0 |  |
| DFID Research for Development Department (R4D) | <https://www.gov.uk/research-for-development-outputs> | energy efficiency intervention | 1230 | 5 | Most of the articles are not relevant to the tpoic |
| ICNL Research Centre | <https://www.icnl.org/> | energy efficiency; residential energy efficiency; energy efficiency intervention; energy saving; energy consumption, energy efficiency measures; | 0 | 0 |  |
| IFPRI | <https://www.ifpri.org/> | energy efficiency; residential energy efficiency; energy efficiency intervention; energy saving; energy consumption, energy efficiency measures; | 5 | 0 | None of the results is relevant to our topic |
| Independent Development Evaluation, AfDB | <https://idev.afdb.org/> | energy efficiency; residential energy efficiency; energy efficiency intervention; energy saving; energy consumption, energy efficiency measures; | 0 | 0 | No result even after searching each term indipendently |
| Innovations for Poverty Action (IPA) | <https://www.poverty-action.org/> | energy efficiency | 0 | 0 |  |
| Inter-American Development Bank Publications | <https://publications.iadb.org/en> | energy efficiency | 7 | 0 | They are not relevant to the topic |
| IRC | <https://www.rescue.org/> | energy efficiency | 245 | 0 | They are not relevant to the topic |
| J-Poverty Action Lab (J-PAL) | <https://www.povertyactionlab.org/> | energy efficiency | 7 | 0 |  |
| Pact-Locus (International Development Coalition) | <https://www.pactworld.org/locus> | energy efficiency | 2 | 1 |  |
| LSE Grantham Research Institute on Climate Change and the Environment | <https://www.lse.ac.uk/granthaminstitute/> | energy efficiency | 54 | 2 |  |
| Mercy Corps | <https://www.mercycorps.org/> | energy efficiency; residential energy efficiency; energy efficiency intervention; energy saving; energy consumption, energy efficiency measures; | 7 | 0 | Combining terms yielded no results, searching independently, only enery saving (5) and energy consumption yielded |
| OECD iLibrary | <https://www.oecd-ilibrary.org/> | energy efficiency; residential energy efficiency; energy efficiency intervention; energy saving; energy consumption, energy efficiency measures | 161 | 3 | All terms were searched independly |
| OpenGrey | <http://www.opengrey.eu/> | energy efficiency; residential energy efficiency; energy efficiency intervention; energy saving; energy consumption, energy efficiency measures; | No exact number available | 0 | Searching key terms independly or combined, none yielded |
| RTI International | <https://www.rti.org/> | energy efficiency; residential energy efficiency; energy efficiency intervention; energy saving; energy consumption, energy efficiency measures; | 0 | 0 |  |
| Samuel Hall (evaluations) | <https://www.samuelhall.org/publications/tag/evaluation> | energy efficiency; | 83 | 0 | None of the results is relevant to our topic |
| The Campbell Collaboration Library | <https://www.campbellcollaboration.org/better-evidence> | energy efficiency; residential energy efficiency; energy efficiency intervention; energy saving; energy consumption, energy efficiency measures; | 2 | 1 | Entering all the key words at once yielded no results, entering one at a time, energyb efficiency(1), and energy efficiency measures(1) |
| Transparency International (TI): | <https://www.transparency.org/en> | energy efficiency; residential energy efficiency; energy efficiency intervention; energy saving; energy consumption, energy efficiency measures; | 4 | 0 | Searched all key words together and independently, energy efficiency,energy saving, energy consumption and energy efficiency measures, all yielded 1 study |
| United Nations Evaluation Group | <http://www.uneval.org/> | energy efficiency; residential energy efficiency; energy efficiency intervention; energy saving; energy consumption, energy efficiency measures; | 2770000 | 0 | The first five pages had no relevant studies |
| USAID Development Clearing House | <https://dec.usaid.gov/dec/home/Default.aspx> | energy efficiency; residential energy efficiency; energy efficiency intervention; energy saving; energy consumption, energy efficiency measures; | 18,782 | 2 | When all key words are searched together, no results are yielded. On independent searching, energy efficiency (8665), residential energy efficiency (139), energy efficiency intervention(11) energy saving(2910), energy consumption (5942)and energy efficiency measures(1115). I searched the first five pages for studies that could be relevant. Only energy efficiency intervention had 2 relevant, all the others had no relevant study |
| World Vision | <https://www.worldvision.org/> | energy efficiency; residential energy efficiency; energy efficiency intervention; energy saving; energy consumption, energy efficiency measures; | 0 | 0 |  |

Below are reported examples of search strategies run in CAB Abstracts, Ensco Discovery, and Econlit:

1. **CAB Abstracts (Ebsco) – Searched 20^th^ November 2020**

S13 S11 AND S12 Limiters - Publication Year: 2000-2020

**1,715**

S12 TI ( ( "quasi experiment*" OR quasi-experiment* OR "random* control* trial*" OR "random* trial*" OR rct* OR ( random* N3 allocat* ) OR evaluat* OR impact* OR assess* OR dif-dif OR "double difference" OR difference-in-difference OR "difference in difference" OR "statistical matching*" OR "propensity score matching" OR "covariate matching" OR "coarsened-exact matching" OR "propensity-weighted" OR "multiple regression" OR "statistical regression" OR "regression discontinuity*" OR "cohort analysis" OR "quantitative method*" OR "program* evaluation" OR "interrupted time series" OR ( before N5 after ) OR ( pre N5 post ) OR ( ( pretest OR "pre test" ) AND ( posttest OR "post test" ) ) OR ( "fixed effect*" N3 ( model OR estimation ) ) OR "instrumental variable*" OR "synthetic control" OR ( ( quantitative OR "comparison group*" OR counterfactual OR "counter factual" OR counter-factual OR experiment* ) N3 ( design OR study OR analysis ) ) ) ) OR AB ( ( "quasi experiment*" OR quasi-experiment* OR "random* control* trial*" OR "random* trial*" OR rct* OR ( random* N3 allocat* ) OR evaluat* OR impact* OR assess* OR dif-dif OR "double difference" OR difference-in-difference OR "difference in difference" OR "statistical matching*" OR "propensity score matching" OR "covariate matching" OR "coarsened-exact matching" OR "propensity-weighted" OR "multiple regression" OR "statistical regression" OR "regression discontinuity*" OR "cohort analysis" OR "quantitative method*" OR "program* evaluation" OR "interrupted time series" OR ( before N5 after ) OR ( pre N5 post ) OR ( ( pretest OR "pre test" ) AND ( posttest OR "post test" ) ) OR ( "fixed effect*" N3 ( model OR estimation ) ) OR "instrumental variable*" OR "synthetic control" OR ( ( quantitative OR "comparison group*" OR counterfactual OR "counter factual" OR counter-factual OR experiment* ) N3 ( design OR study OR analysis ) ) ) ) OR SU ( ( "quasi experiment*" OR quasi-experiment* OR "random* control* trial*" OR "random* trial*" OR rct* OR ( random* N3 allocat* ) OR evaluat* OR impact* OR assess* OR dif-dif OR "double difference" OR difference-in-difference OR "difference in difference" OR "statistical matching*" OR "propensity score matching" OR "covariate matching" OR "coarsened-exact matching" OR "propensity-weighted" OR "multiple regression" OR "statistical regression" OR "regression discontinuity*" OR "cohort analysis" OR "quantitative method*" OR "program* evaluation" OR "interrupted time series" OR ( before N5 after ) OR ( pre N5 post ) OR ( ( pretest OR "pre test" ) AND ( posttest OR "post test" ) ) OR ( "fixed effect*" N3 ( model OR estimation ) ) OR "instrumental variable*" OR "synthetic control" OR ( ( quantitative OR "comparison group*" OR counterfactual OR "counter factual" OR counter-factual OR experiment* ) N3 ( design OR study OR analysis ) ) ) )

2,871,753

S11 S1 OR S2 OR S3 OR S6 OR S7 OR S8 OR S9 OR S10

4,043

S10 TI ( ("energy audit*" or (behavio* N2 chang*) or (information N2 (provid* or provision))) N3 "energy efficien*" N4 (building* or "built environment*" or home or homes or housing or house or houses or household* or residen* or domestic or dwelling* or domicil* or occupan*)) OR AB ( ("energy audit*" or (behavio* N2 chang*) or (information N2 (provid* or provision))) N3 "energy efficien*" N4 (building* or "built environment*" or home or homes or housing or house or houses or household* or residen* or domestic or dwelling* or domicil* or occupan*)) OR SU ( ("energy audit*" or (behavio* N2 chang*) or (information N2 (provid* or provision))) N3 "energy efficien*" N4 (building* or "built environment*" or home or homes or housing or house or houses or household* or residen* or domestic or dwelling* or domicil* or occupan*))

1

S9 TI ( (("fossil fuel*" N3 independen*) or "zero energy" or "green building" or ("whole building*" N3 simulat*) or ("energy productivity" N3 indicator*) or technolog* or (retrofit N2 (level* or program*)) or "air condition*" or HVAC* or "heating ventilation" or lighting or lightbulb* or "light bulb*" or (LED N2 light*)) N4 "energy efficien*" ) OR AB ( (("fossil fuel*" N3 independen*) or "zero energy" or "green building" or ("whole building*" N3 simulat*) or ("energy productivity" N3 indicator*) or technolog* or (retrofit N2 (level* or program*)) or "air condition*" or HVAC* or "heating ventilation" or lighting or lightbulb* or "light bulb*" or (LED N2 light*)) N4 "energy efficien*" ) OR SU ( (("fossil fuel*" N3 independen*) or "zero energy" or "green building" or ("whole building*" N3 simulat*) or ("energy productivity" N3 indicator*) or technolog* or (retrofit N2 (level* or program*)) or "air condition*" or HVAC* or "heating ventilation" or lighting or lightbulb* or "light bulb*" or (LED N2 light*)) N4 "energy efficien*" )

73

S8 TI ( ((furnace* or "energy burden" or infiltration or (electric* N3 "peak demand*")) N3 (replac* or reduc*)) ) OR AB ( ((furnace* or "energy burden" or infiltration or (electric* N3 "peak demand*")) N3 (replac* or reduc*)) ) OR SU ( ((furnace* or "energy burden" or infiltration or (electric* N3 "peak demand*")) N3 (replac* or reduc*)) )

2,289

S7 TI ( ((weatheri* or unweatheri* or replac* or insulat* or upgrad* or "high performance") N3 (window* or door* or attic* or wall or walls or appliance* or "heat pump*" )) ) OR AB ( ((weatheri* or unweatheri* or replac* or insulat* or upgrad* or "high performance") N3 (window* or door* or attic* or wall or walls or appliance* or "heat pump*")) ) OR SU ( ((weatheri* or unweatheri* or replac* or insulat* or upgrad* or "high performance") N3 (window* or door* or attic* or wall or walls or appliance* or "heat pump*")) )

386

S6 S4 AND S5

42

S5 DE "housing" OR DE "cooperative housing" OR DE "dwellings" OR DE "homes" OR DE "public housing" OR DE "rural housing" OR DE "single family housing" OR DE "households" OR DE "housing costs" OR DE "living standards"

45,255

S4 ( DE "electrical energy" OR DE "renewable energy" OR DE "thermal energy" OR DE "energy conservation" OR DE "energy consumption" OR DE "energy sources" ) AND ( DE "efficiency" OR DE "combustion efficiency" OR DE "use efficiency" OR DE "use efficiency" OR DE "thermal efficiency" )

3,503

S3 TI ( ("energy efficien*" N3 (retrofit* or improv* or measur* or program* or intervention*)) ) OR AB ( ("energy efficien*" N3 (retrofit* or improv* or measur* or program* or intervention*)) ) OR SU ( ("energy efficien*" N3 (retrofit* or improv* or measur* or program* or intervention*)) )

1,273

S2 TI ( ((weatheriz* or weatheris*) N3 (building* or "built environment*" or home or homes or housing or house or houses or household* or residen* or domestic or dwelling* or domicil* or occupan*)) ) OR AB ( ((weatheriz* or weatheris*) N3 (building* or "built environment*" or home or homes or housing or house or houses or household* or residen* or domestic or dwelling* or domicil* or occupan*)) ) OR SU ( ((weatheriz* or weatheris*) N3 (building* or "built environment*" or home or homes or housing or house or houses or household* or residen* or domestic or dwelling* or domicil* or occupan*)) )

9

S1 TI (residential N3 "energy efficien*" N3 intervention*) OR AB (residential N3 "energy efficien*" N3 intervention*) OR SU (residential N3 "energy efficien*" N3 intervention*)

3

1. **Ebsco Discovery – Searched 23^rd^ November 2020**

S14 S11 AND S12 Limiters - Date of Publication: **20000101-20211231**

54,141 - **Limited to Repec (1850), Greenfile (1247) & World Bank e-library (35)**

S13 S11 AND S12

62,568

S12 TI ( ( "quasi experiment*" OR quasi-experiment* OR "random* control* trial*" OR "random* trial*" OR rct* OR ( random* N3 allocat* ) OR evaluat* OR impact* OR assess* OR dif-dif OR "double difference" OR difference-in-difference OR "difference in difference" OR "statistical matching*" OR "propensity score matching" OR "covariate matching" OR "coarsened-exact matching" OR "propensity-weighted" OR "multiple regression" OR "statistical regression" OR "regression discontinuity*" OR "cohort analysis" OR "quantitative method*" OR "program* evaluation" OR "interrupted time series" OR ( before N5 after ) OR ( pre N5 post ) OR ( ( pretest OR "pre test" ) AND ( posttest OR "post test" ) ) OR ( "fixed effect*" N3 ( model OR estimation ) ) OR "instrumental variable*" OR "synthetic control" OR ( ( quantitative OR "comparison group*" OR counterfactual OR "counter factual" OR counter-factual OR experiment* ) N3 ( design OR study OR analysis ) ) ) ) OR AB ( ( "quasi experiment*" OR quasi-experiment* OR "random* control* trial*" OR "random* trial*" OR rct* OR ( random* N3 allocat* ) OR evaluat* OR impact* OR assess* OR dif-dif OR "double difference" OR difference-in-difference OR "difference in difference" OR "statistical matching*" OR "propensity score matching" OR "covariate matching" OR "coarsened-exact matching" OR "propensity-weighted" OR "multiple regression" OR "statistical regression" OR "regression discontinuity*" OR "cohort analysis" OR "quantitative method*" OR "program* evaluation" OR "interrupted time series" OR ( before N5 after ) OR ( pre N5 post ) OR ( ( pretest OR "pre test" ) AND ( posttest OR "post test" ) ) OR ( "fixed effect*" N3 ( model OR estimation ) ) OR "instrumental variable*" OR "synthetic control" OR ( ( quantitative OR "comparison group*" OR counterfactual OR "counter factual" OR counter-factual OR experiment* ) N3 ( design OR study OR analysis ) ) ) ) OR SU ( ( "quasi experiment*" OR quasi-experiment* OR "random* control* trial*" OR "random* trial*" OR rct* OR ( random* N3 allocat* ) OR evaluat* OR impact* OR assess* OR dif-dif OR "double difference" OR difference-in-difference OR "difference in difference" OR "statistical matching*" OR "propensity score matching" OR "covariate matching" OR "coarsened-exact matching" OR "propensity-weighted" OR "multiple regression" OR "statistical regression" OR "regression discontinuity*" OR "cohort analysis" OR "quantitative method*" OR "program* evaluation" OR "interrupted time series" OR ( before N5 after ) OR ( pre N5 post ) OR ( ( pretest OR "pre test" ) AND ( posttest OR "post test" ) ) OR ( "fixed effect*" N3 ( model OR estimation ) ) OR "instrumental variable*" OR "synthetic control" OR ( ( quantitative OR "comparison group*" OR counterfactual OR "counter factual" OR counter-factual OR experiment* ) N3 ( design OR study OR analysis ) ) ) )

45,714,338

S11 S1 OR S2 OR S3 OR S6 OR S7 OR S8 OR S9 OR S10

176,223

S10 TI ( ("energy audit*" or (behavio* N2 chang*) or (information N2 (provid* or provision))) N3 "energy efficien*" N4 (building* or "built environment*" or home or homes or housing or house or houses or household* or residen* or domestic or dwelling* or domicil* or occupan*)) OR AB ( ("energy audit*" or (behavio* N2 chang*) or (information N2 (provid* or provision))) N3 "energy efficien*" N4 (building* or "built environment*" or home or homes or housing or house or houses or household* or residen* or domestic or dwelling* or domicil* or occupan*)) OR SU ( ("energy audit*" or (behavio* N2 chang*) or (information N2 (provid* or provision))) N3 "energy efficien*" N4 (building* or "built environment*" or home or homes or housing or house or houses or household* or residen* or domestic or dwelling* or domicil* or occupan*))

162

S9 TI ( (("fossil fuel*" N3 independen*) or "zero energy" or "green building" or ("whole building*" N3 simulat*) or ("energy productivity" N3 indicator*) or technolog* or (retrofit N2 (level* or program*)) or "air condition*" or HVAC* or "heating ventilation" or lighting or lightbulb* or "light bulb*" or (LED N2 light*)) N4 "energy efficien*" ) OR AB ( (("fossil fuel*" N3 independen*) or "zero energy" or "green building" or ("whole building*" N3 simulat*) or ("energy productivity" N3 indicator*) or technolog* or (retrofit N2 (level* or program*)) or "air condition*" or HVAC* or "heating ventilation" or lighting or lightbulb* or "light bulb*" or (LED N2 light*)) N4 "energy efficien*" ) OR SU ( (("fossil fuel*" N3 independen*) or "zero energy" or "green building" or ("whole building*" N3 simulat*) or ("energy productivity" N3 indicator*) or technolog* or (retrofit N2 (level* or program*)) or "air condition*" or HVAC* or "heating ventilation" or lighting or lightbulb* or "light bulb*" or (LED N2 light*)) N4 "energy efficien*" )

10,476

S8 TI ( ((furnace* or "energy burden" or infiltration or (electric* N3 "peak demand*")) N3 (replac* or reduc*)) ) OR AB ( ((furnace* or "energy burden" or infiltration or (electric* N3 "peak demand*")) N3 (replac* or reduc*)) ) OR SU ( ((furnace* or "energy burden" or infiltration or (electric* N3 "peak demand*")) N3 (replac* or reduc*)) )

26,300

S7 TI ( ((weatheri* or unweatheri* or replac* or insulat* or upgrad* or "high performance") N3 (window* or door* or attic* or wall or walls or appliance* or "heat pump*" )) ) OR AB ( ((weatheri* or unweatheri* or replac* or insulat* or upgrad* or "high performance") N3 (window* or door* or attic* or wall or walls or appliance* or "heat pump*")) ) OR SU ( ((weatheri* or unweatheri* or replac* or insulat* or upgrad* or "high performance") N3 (window* or door* or attic* or wall or walls or appliance* or "heat pump*")) )

47,505

S6 S4 AND S5

129

S5 DE "housing" OR DE "cooperative housing" OR DE "dwellings" OR DE "homes" OR DE "public housing" OR DE "rural housing" OR DE "single family housing" OR DE "households" OR DE "housing costs" OR DE "living standards"

1,088,982

S4 ( DE "electrical energy" OR DE "renewable energy" OR DE "thermal energy" OR DE "energy conservation" OR DE "energy consumption" OR DE "energy sources" ) AND ( DE "efficiency" OR DE "combustion efficiency" OR DE "use efficiency" OR DE "use efficiency" OR DE "thermal efficiency" )

11,149

S3 TI ( ("energy efficien*" N3 (retrofit* or improv* or measur* or program* or intervention*)) ) OR AB ( ("energy efficien*" N3 (retrofit* or improv* or measur* or program* or intervention*)) ) OR SU ( ("energy efficien*" N3 (retrofit* or improv* or measur* or program* or intervention*)) )

89,510

S2 TI ( ((weatheriz* or weatheris*) N3 (building* or "built environment*" or home or homes or housing or house or houses or household* or residen* or domestic or dwelling* or domicil* or occupan*)) ) OR AB ( ((weatheriz* or weatheris*) N3 (building* or "built environment*" or home or homes or housing or house or houses or household* or residen* or domestic or dwelling* or domicil* or occupan*)) ) OR SU ( ((weatheriz* or weatheris*) N3 (building* or "built environment*" or home or homes or housing or house or houses or household* or residen* or domestic or dwelling* or domicil* or occupan*)) )

1,159

S1 TI (residential N3 "energy efficien*" N3 intervention*) OR AB (residential N3 "energy efficien*" N3 intervention*) OR SU (residential N3 "energy efficien*" N3 intervention*)

42

1. **Econlit (Ovid) <1886 to November 19, 2020>Searched 23^rd^ November 2020**

1 (residential adj3 "energy efficien*" adj3 intervention*).ti,ab,hw,kw. (1)

2 ((weatheriz* or weatheris*) adj3 (building* or "built environment*" or home or homes or housing or house or houses or household* or residen* or domestic or dwelling* or domicil* or occupan*)).ti,ab,hw,kw. (12)

3 ("energy efficien*" adj3 (retrofit* or improv* or measur* or program* or intervention*)).ti,ab,hw,kw. (1094)

4 ((weatheri* or unweatheri* or replac* or insulat* or upgrad* or "high performance") adj3 (window* or door* or attic* or wall or walls or appliance* or "heat pump*")).ti,ab,hw,kw. (54)

5 ((furnace* or "energy burden" or infiltration or (electric* adj3 "peak demand*")) adj3 (replac* or reduc*)).ti,ab,hw,kw. (6)

6 (("fossil fuel* adj3 independen*" or "zero energy" or "green building" or ("whole building*" adj3 simulat*) or ("energy productivity" adj3 indicator*) or technolog* or (retrofit adj2 (level* or program*)) or "air condition*" or HVAC* or "heating ventilation" or lighting or lightbulb* or "light bulb*" or (LED adj2 light*)) adj4 "energy efficien*").ti,ab,hw,kw. (384)

7 (("energy audit*" or (behavio* adj2 chang*) or (information adj2 (provid* or provision))) adj3 "energy efficien*" adj4 (building* or "built environment*" or home or homes or housing or house or houses or household* or residen* or domestic or dwelling* or domicil* or occupan*)).ti,ab,hw,kw. (1)

8 or/1-7 (1416)

9 ("quasi experiment*" or quasi-experiment* or "random* control* trial*" or "random* trial*" or rct* or (random* adj3 allocat*) or evaluat* or impact* or assess* or dif-dif or "double difference" or difference-in-difference or "difference in difference" or "statistical matching*" or "propensity score matching" or "covariate matching" or "coarsened-exact matching" or "propensity-weighted" or "multiple regression" or "statistical regression" or "regression discontinuity*" or "cohort analysis" or "quantitative method*" or "program* evaluation" or "interrupted time series" or (before adj5 after) or (pre adj5 post) or ((pretest or "pre test") and (posttest or "post test")) or ("fixed effect*" adj3 (model or estimation)) or "instrumental variable*" or "synthetic control" or ((quantitative or "comparison group*" or counterfactual or "counter factual" or counter-factual or experiment*) adj3 (design or study or analysis))).ti,ab,hw,kw. (324972)

10 8 and 9 (654)

11 limit 10 to yr="2000 -Current" (**636**)

# Appendix D. Study information

| Study | Country | Programme | Intervention EEMs | Study design and analysis | Sample | Funding mechanisms |
| --- | --- | --- | --- | --- | --- | --- |
| Adan et al. 2016 | United Kingdom | The Carbon Emission Reduction Target (CERT) and the Community Energy Saving Programme (CESP). Energy Company Obligation (ECO) | Attic/Loft insulation, cavity wall insulation, boiler replacement individually plus all four possible combinations of these EEMs | Regression estimating difference-in-differences | Mix of households^[[1]](#footnote-2)^ | Subsidized |
| Alberini et al. 2016 | United States | Not a programme | Heat pump replacement | Regression with household and time fixed effects | Mix of households | Partially subsidized |
| Alberini et al. 2019 | Ukraine | Not a programme | EEM bundle (attic insulation, basement insulation, cavity wall insulation, double- or triple-glazed windows, new boiler, jackets around hot water pipes) | Regression with household and time fixed effects | Mix of households | Partially subsidized |
| Aydin et al. 2017 | Nether-lands | Meer met Minder | Programme bundle (subsidies for installing REEIs and improving energy label, the energy prediction for home) | Difference-in-differences | Mix of households | Subsidized |
| Beagon et al. 2018 | Ireland | SEAI Better Energy Communities scheme | Programme bundle (cavity wall insulation, gas boiler upgrade, heat boiler replacement by gas boiler, heating controls, lighting replacement, window and door replacement) | Difference-in-differences | Low-income households | Partially subsidized |
| Carranza et al. 2016 | Kyrgyz Republic | Programme name not reported | Up to four CFLs | RCT - Regression with household and time fixed effects | Mix of households | Subsidized |
| Costolanski et al. 2013 | Ethiopia | Programme name not reported | Up to four CFLs | Regression with household and time fixed effects | Mix of households | Subsidized |
| Fowlie et al. 2015 | United States | Weatherization Assistance Programme (WAP) | Programme bundle (most common EEMs were furnace replacement, attic and wall insulation, and infiltration reduction) | RCT - Regression with household and time fixed effects | Low-income households | Subsidized |
| Grimes et al. 2016 | New Zealand | Warm Up New Zealand: Heat Smart (WUNZ:HS) scheme/New Zealand Insulation Fund | Heat pump replacement; Programme bundle (ceiling and under-floor insulation) | Regression with household and time fixed effects | Mix of households | Subsidized |
| Hamilton et al. 2016 | United Kingdom | Not a programme | Attic/loft insulation; Cavity wall insulation; Boiler replacement; Double glazing installation | Regression estimating difference-in-differences | Mix of households | Partially subsidized |
| Howden-Chapman et al. 2007 | New Zealand | Programme name not reported | Programme bundle ("ceiling insulation, insulation around windows and doors, sisalated paper beneath floor joists and a polythene moisture barrier on the ground beneath the house") | RCT - ANCOVA (when energy consumption is the outcome, baseline energy use is a covariate) | Low-income households | Subsidized |
| James et al. 2017 | Australia | South East Councils Climate Change Alliance using funding from Australia's Low Income Energy Efficiency Program | Programme bundle (appliance upgrade, draught sealing, heater/cooler maintenance, heater/cooler upgrade, hot water service insulation, hot water service maintenance, hot water service upgrade, insulation, LED lighting, window treatment, and zoning"; or  Programme bundle + behaviour change intervention ("one-on-one meeting to discuss motivations and choice of energy actions; follow-up meeting to discuss motivations and choice of energy actions; group meeting to discuss energy actions taken, challenges, and to share learnings; installation of a Watts Clever EW4500 in-home-display; installation of an EMS Ecofront energy monitor in-home-display") | RCT and Difference-in-differences | Low-income households | Subsidized |
| Liang et al. 2017 | United States | Energize Phoenix programme | Programme bundle (subsidies for five possible retrofit components: air conditioner upgrades, air sealing, duct sealing, insulation, and shade screens; homeowners chose which components) | Regression with household and time fixed effects | Mix of households | Subsidized |
| Maher et al. 2013 | United States | Gainesville Regional Utility retrofit rebate programmes | Attic/loft insulation; Super SEER air conditioning | Regression with household and time fixed effects | Mix of households | Partially subsidized |
| Scheer et al. 2013 | Ireland | SEAI Better Energy Communities scheme | Programme bundle (cavity wall insulation, ceiling insulation, new boiler, heating controls, external insulation, dry lining) | Difference-in-differences | Mix of households | Partially subsidized |
| Suter et al. 2013 | United States | Not a programme | Attic/loft insulation (with and without information) | RCT - Regression with household and time fixed effects | Rented undergraduate housing | Subsidized |

*Notes.* Some of the studies that did not evaluate a specific programme (Hamilton et al. 2016; Alberini et al. 2016; Alberini et al. 2019) included some households who participated in a programme as well as households that did not.

# Appendix E. Appraised risk of bias for each study on each criterion

*Table E.1 Randomized controlled trials*

| Study | Valid random assignment mechanism | Free from selection bias | Free from confound-ing | Free from deviations from intended interven-tions | Free from perform-ance bias | Free from outcome measure-ment bias | Free from analysis, reporting bias | Free from other bias | Overall risk of bias level |
| --- | --- | --- | --- | --- | --- | --- | --- | --- | --- |
| Howden-Chapman et al. (2007) | Yes | Probably yes | Yes | Probably yes | Probably yes | Probably yes | Yes | Yes | Low |
| James and Ambrose (2017) | Probably no | Probably no | Probably no | Probably yes | Yes | No | Probably yes | Yes | High |
| Suter et al. 2013 | Probably yes | Yes | Probably yes | Probably yes | Probably yes | Yes | Probably yes | Yes | Low |
| Fowlie et al. 2018 | Probably yes | Yes | Yes | Yes | Yes | Yes | Probably yes | Yes | Low |
| Carranza and Meeks (2016) | Probably yes | Unclear | Yes | Yes | Yes | Yes | Probably yes | Yes | Some concerns |

*Table E.2 Quasi-experimental designs*

| Study | Free from selection bias | Free from confounding | Free from deviations from intended interventions | Free from performance bias | Free from outcome measurement bias | Free from analysis, reporting bias | Free from other bias | Risk of bias level |
| --- | --- | --- | --- | --- | --- | --- | --- | --- |
| Adan et al. 2016 | Probably yes | Yes | Yes | Yes | Yes | Probably yes | Yes | Low |
| Alberiniet al. 2016 | No | No | Yes | Yes | Yes | Yes | Yes | High |
| Alberini et al. 2019 | No | Unclear | Yes | Yes | Yes | Yes | Yes | High |
| Aydin et al. 2017 | No | No | Yes | Yes | Yes | Yes | Yes | High |
| Beagon et al. 2018 | No | Unclear | Yes | Yes | Yes | Probably yes | Yes | High |
| Costolanski et al. 2013 | No | Probably no | Yes | Yes | Yes | Probably yes | Yes | High |
| Grimes et al. 2016 | Probably yes | Yes | Yes | Yes | Yes | Yes | Yes | Low |
| Hamilton et al. 2016 | No | Unclear | Probably yes | Yes | Yes | Yes | Yes | High |
| Liang et al. 2018 | Probably yes | Probably no | Probably yes | Yes | Yes | Yes | Yes | High |
| Maher 2013 | Unclear | Unclear | Yes | Yes | Yes | Yes | Yes | Some concerns |
| Scheer et al. 2013 | No | No | Probably yes | Yes | Yes | Yes | Yes | High |

# Appendix F. Heterogeneity statistics

In meta-analysis, heterogeneity typically refers to how effects vary by populations and interventions (that is, how consistent effects are across populations and interventions). For each meta-analysis presented in this report, Table F.1 reports the following heterogeneity statistics:

- Average effect size (Hedges’ g)
- Q-statistic (sum of squared deviations on standardized scale)
- degrees of freedom (number of studies - 1)
- p-value (the null hypothesis is that there is no variation in true effects)
- I^2^ (ratio of true effect variance to total variance)
- T (standard deviation of true effects; square root of T^2^, the estimated variance of true effects)

*Table F.1. Heterogeneity statistics*

| **Type of REEI** | **Hedges’ g** | **Q** | **df** | **p-value** | **I^2^** | **T** |
| --- | --- | --- | --- | --- | --- | --- |
| Compact fluorescent light bulbs (CFLs) | -0.29 | 14.21 | 1 | .000 | 85.81 | 0.128 |
| Attic insulation | -0.04 | 50.04 | 3 | .000 | 95.43 | 0.043 |
| Electric heat pumps | -0.11 | 16.31 | 1 | .000 | 87.56 | 0.207 |
| EEM bundle | -0.36 | 249.86 | 6 | .000 | 94.57 | 0.195 |
| EEM bundle (low-income households) | -0.16 | 1.46 | 2 | .483 | 0 | 0 |
| EEM bundle (European Union) | -0.61 | 2.69 | 2 | .261 | 0.05 | 0.001 |
| EEM bundle (low risk of bias) | -0.12 | 0.58 | 1 | .445 | 0 | 0 |
| EEM bundle (audit only) | -0.42 | 247.32 | 4 | .000 | 95.59 | 0.185 |

The p-value presented in Table F.1 indicate there is statistically significant variation in true effects for most meta-analyses, consistent with REEI impacts varying by study population and intervention. The I^2^ for these analyses are always above 85, indicating that most variance in impacts is due to variance in true effects not sampling error. The remaining three analyses that do not indicate statistically significant heterogeneity include only two or three studies and might be underpowered to detect variation in true effects (Borenstein et al. 2009; p. 113).

# Appendix G. Model and estimator sensitivity

This appendix presents, for each meta-analysis, the average impact and standard error estimated using either a restricted maximum likelihood estimator (Table G.1) or a fixed-effects model (Table G2).

The restricted maximum likelihood estimator is recommended by Viechtbauer (2005), and for this model we also report T^2^, an estimate of the true impact variation.

*Table G.1 Impacts of REEIs, estimated using restricted maximum likelihood*

| **Type of REEI** | **Average impact** | **Standard error** | **T^2^** |
| --- | --- | --- | --- |
| Compact fluorescent light bulbs (CFLs) | -0.28 | 0.14 | 0.036 |
| Attic insulation | -0.05 | 0.03 | 0.003 |
| Electric heat pumps | -0.12 | 0.22 | 0.093 |
| EEM bundle | -0.42 | 0.10 | 0.043 |

We believe the random-effects model that was used to estimate the primary statistics is more appropriate because (1) the true impact likely varies across studies due to variation in interventions, climates, and populations, and (2) we are interested in making inferences about the population of studies (Borenstein et al. 2011). However, because the number of studies included in each meta-analysis is relatively small—and thus the random-effects estimates have poor precision—we also report statistics estimated using a fixed-effects model in this appendix. Because the fixed-effects model assumes that the impact for all studies is identical and study impacts are weighted by precision, the fixed-effects average impact is heavily influenced by the studies with the largest samples.

*Table G.2 Fixed-effects meta-analysis model*

| **Type of REEI** | **Average impact** | **Standard error** |
| --- | --- | --- |
| Compact fluorescent light bulbs (CFLs) | -0.36 | 0.03 |
| Attic insulation | -0.016 | 0.004 |
| Electric heat pumps | 0.08 | 0.01 |
| EEM bundle | -0.24 | 0.01 |

# Appendix H. Supplemental findings

# *Table H.1 Study-reported impacts on energy consumption*

| Study | Annual impact per household in KWh  (percentage change in consumption) | Hedges’ g  (SE) |
| --- | --- | --- |
| ***Boiler replacement*** | | |
| ***Hamilton (2016)*** | ***-1059 kilowatt hours*** | ***-0.05 (0.006)*** |
| ***Cavity wall insulation*** | | |
| ***Hamilton (2016)*** | ***-1047 kilowatt hours*** | ***-0.08 (0.006)*** |
| ***Insulation (any type)*** | | |
| ***Alberini (2019)*** | ***(-2 percentage points)*** | ***-0.05 (0.13)*** |
| ***Grimes (2016)*** | ***-139 kilowatt hours*** | ***-0.21 (0.01)*** |
| ***Insulation (any type) and windows (double- or triple-glazed)*** | | |
| ***Alberini (2019)*** | ***(-5 percentage points)*** | ***-0.15 (0.10)*** |
| ***Thermostat*** | | |
| ***Suter (2013)*** | ***-77 kilowatt hours*** | ***-0.32 (0.40)*** |
| ***Windows (double-glazed)*** | | |
| ***Hamilton (2016)*** | ***-12 kilowatt hours*** | ***-0.00 (0.01)*** |

# *Table H.2 Study-reported impacts on indoor temperature*

| Study | Annual impact per household in KWh  (percentage change in consumption) | Hedges’ g  (SE) |
| --- | --- | --- |
| ***Thermostat*** | | |
| ***Suter (2013)*** | ***-0.09 degrees*** | ***-0.06 (0.40)*** |

Notes. *Notes.* Suter et al. impacts were reported in Fahrenheit and converted to Celsius by multiplying by 5/9.

# Appendix I. Supplemental analysis of EEMs involving an audit

The primary analysis of EEM bundles involved all seven studies that included an EEM bundle. This appendix presents the forest plot for a subset of those studies, the five studies examining an EEMs bundle where the authors reported there was an energy audit prior to installation.

*Figure I.1. Impacts of EEM bundles with audit, by climate*


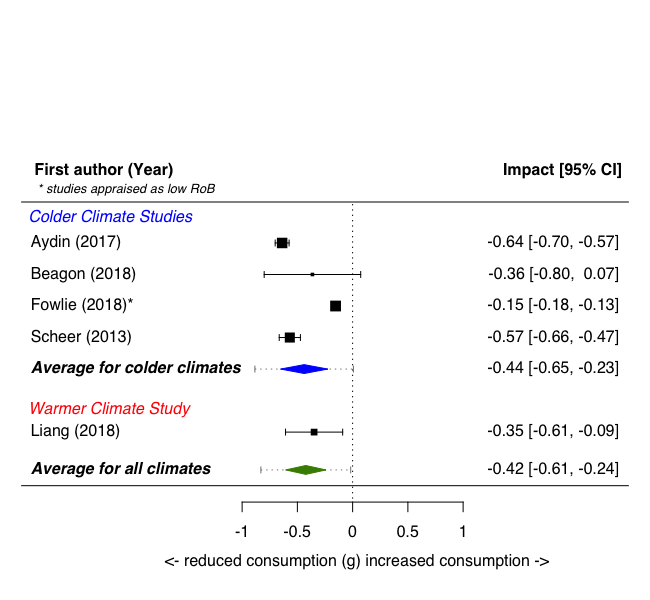


For individual studies, the the rightmost column and the horizontal lines indicate the 95% confidence intervals (95% of the time this interval will capture the actual impact). For the average impacts, the confidence interval is displayed in the rightmost column and represented by the width of the diamond, while the dashed horizontal lines indicate the prediction interval (the range in which the impact will fall for 95% of populations).

# Appendix J. Meta-analysis statistics with individual studies removed

| **Study Removed** | **Average** | **SE** | **Q** | **Tau** | **I^2^** |
| --- | --- | --- | --- | --- | --- |
| *Attic insulation* | | | | | |
| Adan (2016) | -0.26 | 0.22 | 32.37 | 0.35 | 99.53% |
| Hamilton (2016) | -0.06 | 0.03 | 18.80 | 0.03 | 66.34% |
| Maher (2013) | -0.26 | 0.24 | 35.71 | 0.39 | 99.96% |
| Suter (2016) | -0.04 | 0.03 | 40.24 | 0.04 | 96.65% |
| *EEM bundles that vary by household* | | | | | |
| Alberini (2019) | -0.40 | 0.09 | 289.77 | 0.04 | 94.88% |
| Aydin (2017) | -0.29 | 0.08 | 83.87 | 0.03 | 87.42% |
| Beagon (2018) | -0.35 | 0.09 | 290.76 | 0.04 | 96.05% |
| Fowlie (2018) | -0.41 | 0.09 | 36.07 | 0.03 | 85.72% |
| Howden-Chapman (2007) | -0.36 | 0.10 | 291.05 | 0.05 | 96.08% |
| Liang (2018) | -0.35 | 0.10 | 290.05 | 0.05 | 96.15% |
| Scheer (2013) | -0.31 | 0.09 | 232.45 | 0.04 | 94.15% |

SE=Standard error of the average impact estimate

# *Notes.* Estimation excluding Adan et al., Hamilton et al., and Maher led to a warning that the Fisher scoring algorithm may have gotten stuck at a local maximum, and that tau2 was set to 0.

1. Mix households refer to a group of households of any income level. Some studies targeted low-income households. [↑](#footnote-ref-2)
